# Genome-wide association study meta-analysis of dizygotic twinning illuminates genetic regulation of female fecundity

**DOI:** 10.1093/humrep/dead247

**Published:** 2023-12-05

**Authors:** Hamdi Mbarek, Scott D Gordon, David L Duffy, Nikki Hubers, Sally Mortlock, Jeffrey J Beck, Jouke-Jan Hottenga, René Pool, Conor V Dolan, Ky’Era V Actkins, Zachary F Gerring, Jenny Van Dongen, Erik A Ehli, William G Iacono, Matt Mcgue, Daniel I Chasman, C Scott Gallagher, Samantha L P Schilit, Cynthia C Morton, Guillaume Paré, Gonneke Willemsen, David C Whiteman, Catherine M Olsen, Catherine Derom, Robert Vlietinck, Daniel Gudbjartsson, Lisa Cannon-Albright, Eva Krapohl, Robert Plomin, Patrik K E Magnusson, Nancy L Pedersen, Pirro Hysi, Massimo Mangino, Timothy D Spector, Teemu Palviainen, Yuri Milaneschi, Brenda W Penninnx, Adrian I Campos, Ken K Ong, John R B Perry, Cornelis B Lambalk, Jaakko Kaprio, Ísleifur Ólafsson, Karine Duroure, Céline Revenu, Miguel E Rentería, Loic Yengo, Lea Davis, Eske M Derks, Sarah E Medland, Hreinn Stefansson, Kari Stefansson, Filippo Del Bene, Bruno Reversade, Grant W Montgomery, Dorret I Boomsma, Nicholas G Martin

**Affiliations:** Department of Biological Psychology, Netherlands Twin Register, Vrije Universiteit, Amsterdam, The Netherlands; Qatar Genome Program, Qatar Foundation, Doha, Qatar; Amsterdam Reproduction and Development Institute, Amsterdam, The Netherlands; QIMR Berghofer Medical Research Institute, Brisbane, QLD, Australia; QIMR Berghofer Medical Research Institute, Brisbane, QLD, Australia; Department of Biological Psychology, Netherlands Twin Register, Vrije Universiteit, Amsterdam, The Netherlands; Amsterdam Reproduction and Development Institute, Amsterdam, The Netherlands; Institute of Molecular Bioscience, University of Queensland, Brisbane, QLD, Australia; Avera Institute for Human Genetics, Avera McKennan Hospital and University Health Center, Sioux Falls, SD, USA; Department of Biological Psychology, Netherlands Twin Register, Vrije Universiteit, Amsterdam, The Netherlands; Department of Biological Psychology, Netherlands Twin Register, Vrije Universiteit, Amsterdam, The Netherlands; Department of Biological Psychology, Netherlands Twin Register, Vrije Universiteit, Amsterdam, The Netherlands; Vanderbilt Genetics Institute, Vanderbilt University, Nashville, TN, USA; QIMR Berghofer Medical Research Institute, Brisbane, QLD, Australia; Department of Biological Psychology, Netherlands Twin Register, Vrije Universiteit, Amsterdam, The Netherlands; Amsterdam Reproduction and Development Institute, Amsterdam, The Netherlands; Avera Institute for Human Genetics, Avera McKennan Hospital and University Health Center, Sioux Falls, SD, USA; Department of Psychology, University of Minnesota, Minneapolis, MN, USA; Department of Psychology, University of Minnesota, Minneapolis, MN, USA; Harvard Medical School, Harvard University, Boston, MA, USA; Brigham and Women’s Hospital, Boston, MA, USA; Harvard Medical School, Harvard University, Boston, MA, USA; Harvard Medical School, Harvard University, Boston, MA, USA; Brigham and Women’s Hospital, Boston, MA, USA; Harvard Medical School, Harvard University, Boston, MA, USA; Brigham and Women’s Hospital, Boston, MA, USA; Population Health Research Institute, McMaster University, Hamilton, ON, Canada; Department of Biological Psychology, Netherlands Twin Register, Vrije Universiteit, Amsterdam, The Netherlands; Qatar Genome Program, Qatar Foundation, Doha, Qatar; QIMR Berghofer Medical Research Institute, Brisbane, QLD, Australia; University of Leuven, Leuven, Belgium; University of Leuven, Leuven, Belgium; deCODE Genetics, Reykjavik, Iceland; University of Utah, Salt Lake City, UT, USA; Medical Research Council Social, Genetic and Developmental Psychiatry Centre, Institute of Psychiatry, Psychology & Neuroscience, King’s College London, London, UK; Statistical Sciences & Innovation, UCB Biosciences GmbH, Monheim, Germany; Medical Research Council Social, Genetic and Developmental Psychiatry Centre, Institute of Psychiatry, Psychology & Neuroscience, King’s College London, London, UK; Department of Medical Epidemiology and Biostatistics, Karolinska Institutet, Stockholm, Sweden; Department of Medical Epidemiology and Biostatistics, Karolinska Institutet, Stockholm, Sweden; Department of Twin Research & Genetic Epidemiology, King’s College London, London, UK; Department of Twin Research & Genetic Epidemiology, King’s College London, London, UK; NIHR Biomedical Research Centre at Guy’s and St Thomas’ Foundation Trust, London, UK; Department of Twin Research & Genetic Epidemiology, King’s College London, London, UK; Institute for Molecular Medicine Finland FIMM, University of Helsinki, Helsinki, Finland; Department of Psychiatry, EMGO Institute for Health and Care Research, Vrije Universiteit, Amsterdam, The Netherlands; Department of Psychiatry, EMGO Institute for Health and Care Research, Vrije Universiteit, Amsterdam, The Netherlands; QIMR Berghofer Medical Research Institute, Brisbane, QLD, Australia; Institute of Molecular Bioscience, University of Queensland, Brisbane, QLD, Australia; MRC Epidemiology Unit, University of Cambridge School of Clinical Medicine, Institute of Metabolic Science, Cambridge, UK; MRC Epidemiology Unit, University of Cambridge School of Clinical Medicine, Institute of Metabolic Science, Cambridge, UK; Amsterdam Reproduction and Development Institute, Amsterdam, The Netherlands; Amsterdam University Medical Centers Location VU Medical Center, Amsterdam, The Netherlands; Institute for Molecular Medicine Finland FIMM, University of Helsinki, Helsinki, Finland; Department of Clinical Biochemistry, National University Hospital of Iceland, Reykjavik, Iceland; Sorbonne Université, INSERM, CNRS, Institut de la Vision, Paris, France; Sorbonne Université, INSERM, CNRS, Institut de la Vision, Paris, France; QIMR Berghofer Medical Research Institute, Brisbane, QLD, Australia; Institute of Molecular Bioscience, University of Queensland, Brisbane, QLD, Australia; Vanderbilt Genetics Institute, Vanderbilt University, Nashville, TN, USA; QIMR Berghofer Medical Research Institute, Brisbane, QLD, Australia; QIMR Berghofer Medical Research Institute, Brisbane, QLD, Australia; deCODE Genetics, Reykjavik, Iceland; deCODE Genetics, Reykjavik, Iceland; Sorbonne Université, INSERM, CNRS, Institut de la Vision, Paris, France; Genome Institute of Singapore, Laboratory of Human Genetics & Therapeutics, A*STAR, Singapore, Singapore; Smart-Health Initiative, BESE, KAUST, Thuwal, Saudi Arabia; Institute of Molecular Bioscience, University of Queensland, Brisbane, QLD, Australia; Department of Biological Psychology, Netherlands Twin Register, Vrije Universiteit, Amsterdam, The Netherlands; Amsterdam Reproduction and Development Institute, Amsterdam, The Netherlands; QIMR Berghofer Medical Research Institute, Brisbane, QLD, Australia

**Keywords:** dizygotic twinning, genome-wide association analysis, female fecundity, fertility, genetics

## Abstract

**STUDY QUESTION:**

Which genetic factors regulate female propensity for giving birth to spontaneous dizygotic (DZ) twins?

**SUMMARY ANSWER:**

We identified four new loci, *GNRH1*, *FSHR*, *ZFPM1*, and *IPO8*, in addition to previously identified loci, *FSHB* and *SMAD3*.

**WHAT IS KNOWN ALREADY:**

The propensity to give birth to DZ twins runs in families. Earlier, we reported that *FSHB* and *SMAD3* as associated with DZ twinning and female fertility measures.

**STUDY DESIGN, SIZE, DURATION:**

We conducted a genome-wide association meta-analysis (GWAMA) of mothers of spontaneous dizygotic (DZ) twins (8265 cases, 264 567 controls) and of independent DZ twin offspring (26 252 cases, 417 433 controls).

**PARTICIPANTS/MATERIALS, SETTING, METHODS:**

Over 700 000 mothers of DZ twins, twin individuals and singletons from large cohorts in Australia/New Zealand, Europe, and the USA were carefully screened to exclude twins born after use of ARTs. Genetic association analyses by cohort were followed by meta-analysis, phenome wide association studies (PheWAS), *in silico* and *in vivo* annotations, and Zebrafish functional validation.

**MAIN RESULTS AND THE ROLE OF CHANCE:**

This study enlarges the sample size considerably from previous efforts, finding four genome-wide significant loci, including two novel signals and a further two novel genes that are implicated by gene level enrichment analyses. The novel loci, *GNRH1* and *FSHR*, have well-established roles in female reproduction whereas *ZFPM1* and *IPO8* have not previously been implicated in female fertility. We found significant genetic correlations with multiple aspects of female reproduction and body size as well as evidence for significant selection against DZ twinning during human evolution. The 26 top single nucleotide polymorphisms (SNPs) from our GWAMA in European-origin participants weakly predicted the crude twinning rates in 47 non-European populations (*r* = 0.23 between risk score and population prevalence, s.e. 0.11, 1-tail *P* = 0.058) indicating that genome-wide association studies (GWAS) are needed in African and Asian populations to explore the causes of their respectively high and low DZ twinning rates. *In vivo* functional tests in zebrafish for *IPO8* validated its essential role in female, but not male, fertility. In most regions, risk SNPs linked to known expression quantitative trait loci (eQTLs). Top SNPs were associated with in vivo reproductive hormone levels with the top pathways including hormone ligand binding receptors and the ovulation cycle.

**LARGE SCALE DATA:**

The full DZT GWAS summary statistics will made available after publication through the GWAS catalog (https://www.ebi.ac.uk/gwas/).

**LIMITATIONS, REASONS FOR CAUTION:**

Our study only included European ancestry cohorts. Inclusion of data from Africa (with the highest twining rate) and Asia (with the lowest rate) would illuminate further the biology of twinning and female fertility.

**WIDER IMPLICATIONS OF THE FINDINGS:**

About one in 40 babies born in the world is a twin and there is much speculation on why twinning runs in families. We hope our results will inform investigations of ovarian response in new and existing ARTs and the causes of female infertility.

**STUDY FUNDING/COMPETING INTEREST(S):**

Support for the Netherlands Twin Register came from the Netherlands Organization for Scientific Research (NWO) and The Netherlands Organization for Health Research and Development (ZonMW) grants, 904-61-193, 480-04-004, 400-05-717, Addiction-31160008, 911-09-032, Biobanking and Biomolecular Resources Research Infrastructure (BBMRI.NL, 184.021.007), Royal Netherlands Academy of Science Professor Award (PAH/6635) to DIB, European Research Council (ERC-230374), Rutgers University Cell and DNA Repository (NIMH U24 MH068457-06), the Avera Institute, Sioux Falls, South Dakota (USA) and the National Institutes of Health (NIH R01 HD042157-01A1) and the Genetic Association Information Network (GAIN) of the Foundation for the National Institutes of Health and Grand Opportunity grants 1RC2 MH089951. The QIMR Berghofer Medical Research Institute (QIMR) study was supported by grants from the National Health and Medical Research Council (NHMRC) of Australia (241944, 339462, 389927, 389875, 389891, 389892, 389938, 443036, 442915, 442981, 496610, 496739, 552485, 552498, 1050208, 1075175). L.Y. is funded by Australian Research Council (Grant number DE200100425). The Minnesota Center for Twin and Family Research (MCTFR) was supported in part by USPHS Grants from the National Institute on Alcohol Abuse and Alcoholism (AA09367 and AA11886) and the National Institute on Drug Abuse (DA05147, DA13240, and DA024417). The Women’s Genome Health Study (WGHS) was funded by the National Heart, Lung, and Blood Institute (HL043851 and HL080467) and the National Cancer Institute (CA047988 and UM1CA182913), with support for genotyping provided by Amgen. Data collection in the Finnish Twin Registry has been supported by the Wellcome Trust Sanger Institute, the Broad Institute, ENGAGE—European Network for Genetic and Genomic Epidemiology, FP7-HEALTH-F4-2007, grant agreement number 201413, National Institute of Alcohol Abuse and Alcoholism (grants AA-12502, AA-00145, AA-09203, AA15416, and K02AA018755) and the Academy of Finland (grants 100499, 205585, 118555, 141054, 264146, 308248, 312073 and 336823 to J. Kaprio). TwinsUK is funded by the Wellcome Trust, Medical Research Council, Versus Arthritis, European Union Horizon 2020, Chronic Disease Research Foundation (CDRF), Zoe Ltd and the National Institute for Health Research (NIHR) Clinical Research Network (CRN) and Biomedical Research Centre based at Guy’s and St Thomas’ NHS Foundation Trust in partnership with King’s College London. For NESDA, funding was obtained from the Netherlands Organization for Scientific Research (Geestkracht program grant 10000-1002), the Center for Medical Systems Biology (CSMB, NVVO Genomics), Biobanking and Biomolecular Resources Research Infrastructure (BBMRI-NL), VU University’s Institutes for Health and Care Research (EMGO+) and Neuroscience Campus Amsterdam, University Medical Center Groningen, Leiden University Medical Center, National Institutes of Health (NIH, ROI D0042157-01A, MH081802, Grand Opportunity grants 1 RC2 Ml-1089951 and IRC2 MH089995). Part of the genotyping and analyses were funded by the Genetic Association Information Network (GAIN) of the Foundation for the National Institutes of Health. Computing was supported by BiG Grid, the Dutch e-Science Grid, which is financially supported by NWO. Work in the Del Bene lab was supported by the Programme Investissements d’Avenir IHU FOReSIGHT (ANR-18-IAHU-01). C.R. was supported by an EU Horizon 2020 Marie Skłodowska-Curie Action fellowship (H2020-MSCA-IF-2014 #661527). H.S. and K.S. are employees of deCODE Genetics/Amgen. The other authors declare no competing financial interests.

**TRIAL REGISTRATION NUMBER:**

N/A.

## Introduction

Spontaneous dizygotic (DZ) twinning frequently runs in families and varies widely between major ancestry groups, from as low as 2/1000 in individuals with East Asian ancestries to as high as 20/1000 in individuals with West African ancestries. In contrast, monozygotic (MZ) twinning occurs at a somewhat constant rate of about 4/1000 births and, with some rare exceptions, does not appear to run in families (Bulmer, 1970).

Multiple births are associated with considerable risk to maternal and infant health (Santana *et al.*, 2016, 2018; Monden and Smits, 2017) and the factors regulating DZ twinning frequency and their relationship to fertility and reproductive aging are still poorly understood, both at the genetic and environmental level. Two well-established factors influencing DZ twinning are maternal age and number of previous children. Increasing maternal age is one of the reasons that spontaneous DZ rates have increased in recent decades. Compared to mothers aged 18 years, the DZ rate in mothers aged 35 is about 4-fold higher but it declines rapidly thereafter due to ovarian depletion (Bulmer, 1970).

We previously reported the first two genetic loci associated with being a mother of spontaneous dizygotic (DZ) twins (MoDZT), based on a genome-wide association meta-analysis (GWAMA) of 1980 cases and 12 953 controls (Mbarek *et al.*, 2016a,b, 2019). We found variants upstream of *FSHB*, the structural locus for FSH beta subunit, and in the first intron of *SMAD3*, which regulates the ovarian response to FSH. This was replicated with gene-based testing of ‘being a DZ twin’ in the UK Biobank (UKBB) (Mbarek *et al.*, 2019). Here we have substantially expanded our discovery sample to 8265 MoDZT plus 264 567 controls and 26 252 DZ twin individuals plus 417 433 controls from European ancestry from Australia/New Zealand (ANZ), Europe, and the USA (Table 1 and Supplementary Data File S1).

**Table 1.** Case and control numbers by cohort.

Cohort—mothers of DZ twins	Cases	Controls	Total
Netherlands (NTR)	1186	5746	6932
Australia & NZ (QIMR)	3273	24 009	27 282
USA (MCTFR)	568	1862	2430
USA (WGHS)	361	20 183	20 544
Iceland (deCODE) born < 1991	2877	212 767	215 644
**Total MoDZTs**	**8265**	**264** **567**	**272** **832**
UK Biobank (likely a DZ twin)	8962	409 591	418 553
Sweden (being a DZ twin)	5373	1404	6777
Finland (being a DZ twin)	6021	2167	8188
TEDS—UK (being a DZ twin)	4125	2565	6690
UK Twins (being a DZ twin)	1771	1706	3477
**Total being a twin**	**26252**	**417** **433**	**443** **685**

For NTR, QIMR, MCTFR, WGHS, and deCODE cases are mothers of spontaneous DZ twins (MoDZTs); for UK Biobank a ‘case’ is a participant who checked ‘part of a multiple birth’, born before 1970 (ergo no ART) and without an MZ co-twin also enrolled in UK Biobank. For Sweden, Finland, TEDS and UK Twins, a case is a DZ twin individual (one per pair, selected randomly). For controls, all individuals are unrelated.

There has been a large increase in DZ twinning following the widespread adoption of assisted reproductive techniques (ART) in the 1980s (Monden *et al.*, 2021). In our previous work (Mbarek *et al.*, 2016b), we showed the sensitivity of the phenotype definition to contamination with DZ twins conceived after ART, with as few as 10% ART cases entirely ablating the association signals. Therefore, we selected only DZ twin pregnancies from before the year of introduction of ART in each country (1970–1985) if this information was not available for twins or their mothers. In cohorts with individual information on use of ART, we carefully filtered phenotypic data for non-spontaneous twin pregnancies.

## Materials and methods

### Phenotype collection

#### Australia (including New Zealand, Belgium and Utah)

Samples (3273 cases and 24 009 controls) were collected by the Queensland Institute of Medical Research (QIMR Berghofer; QIMR), from a number of studies which collected DNA in twin families since around 1990. The collection was augmented by the QIMR Twinning Genetics (TG) study which recruited mothers of dizygotic (DZ) twins (MoDZT) from DZ-twin-rich families (Montgomery *et al.*, 2001; Painter *et al.*, 2010); for these, zygosity was mainly established by surveys and telephone interviews. To restrict analysis as much as possible to mothers of spontaneous twins, if the twins had been born after about 1980 (before 1980, ART was rare) and the mother was still contactable (75%), she was asked by email or phone about ART. When her twins were of same sex, questions to establish zygosity were also asked (Martin and Martin, 1975). Nevertheless, the sample may still contain some ART twins and these will diminish genome-wide association studies (GWAS) signals (see above). Full details of our recruitment strategy are given in Gordon *et al.* (2023) and in Supplementary Data File S1. As northern European-origin controls, we included (i) individuals with available genotyping from twin studies, who were not known to be genetically related to a mother of DZ twins; (ii) the QIMR Berghofer QSkin Study (Olsen *et al.*, 2012) which is an unselected sample of Queenslanders aged 40–70 randomly contacted via the electoral roll (as voter registration is compulsory in Australia) (Supplementary Data File S1).

#### Netherlands Twin Register (NTR)

The NTR sample consisted of 1186 cases (MoDZT) and 5746 controls from the NTR (3377 participants) and the Netherlands Study of Depression and Anxiety (NESDA; 2369 participants). NTR participants were ascertained by the presence of liveborn twins or triplets in the family and include their family members. Twins are born in all strata of society and NTR represents a general sample from the Dutch population. NESDA is a longitudinal study of depression and anxiety disorders whose participants were recruited from the general population, mental health organizations and general practices (Boomsma *et al.*, 2008). Zygosity was confirmed by DNA genotyping. Data on mode of pregnancy were available from surveys sent out to mothers of twins or to parents upon registration of young twins, and from telephone interviews as part of a project on DZ twinning (Hoekstra *et al.*, 2008). A comparison of survey data to hospital records showed that mothers can accurately report on the mode of conception of their twins (van Beijsterveldt *et al.*, 2008). Participants were excluded if they reported ART on one or more occasions. If no reports on mode of pregnancy were available, data were excluded unless the twins were born prior to 1985 (Supplementary Data File S1).

#### Minnesota Center for Twin and Family Research

All subjects in this sample were independently ascertained through vital records of the State of Minnesota in an effort to construct a population-based twin registry (Iacono *et al.*, 1999; Miller *et al.*, 2012). The current study included 568 mothers of DZ twins and 1862 control subjects who were the parents of MZ twins from 1062 families, including 800 complete parental pairs, 203 mothers, and 59 fathers. Most twins were born in the 1970s or early 1980s when, even though fertility treatment was available in the USA, it was expensive and few had access to it. Genotyping was population based and independent of phenotypes other than twinning. About 92% of the registry, and 100% of both case and control samples, are of primarily European ancestry.

#### Boston study WGHS

The Women’s Genome Health Study (WGHS) is a population-based, prospective cohort of initially healthy, female North American health care professionals at least 45 years old at baseline, representing participants in the Women’s Health Study (WHS) who provided a blood sample at baseline and consented for blood-based analyses. The WHS was a 2 × 2 trial beginning in 1992–1994 of vitamin E and low dose aspirin in prevention of cancer and cardiovascular disease with about 10 years of follow-up. Since the end of the trial, follow-up has continued in observational mode. Additional information related to health and lifestyle were collected by questionnaire throughout the WHS trial and continuing observational follow-up. The WGHS cohort has been described previously (Ridker *et al.*, 2008). A total of 361 mothers of DZ twins and 20 183 controls from the WGHS were included in the current study.

#### Iceland (deCODE)

Mothers of twins or other multiples (‘twins’) were selected from among those taking part in deCODE’s genetic studies based on a nation-wide genealogical database. To increase the proportion of these mothers of twins who were mothers of DZ twins, twins who had been genotyped and shown to be MZ were not used to identify mothers. Control subjects were individuals participating in deCODE’s genetic research, from which both mothers of twins and the mothers’ first-degree relatives had been removed. In total, the deCODE sample included in the current study consisted of 2877 mothers of DZ twins and 212 767 controls.

#### TwinsUK study

The TwinsUK Study recruited monozygotic (MZ) and dizygotic (DZ) twin pairs from the TwinsUK adult twin registry, a group designed to study the heritability and genetics of age-related diseases. These twins were recruited from the general population through national media campaigns in the UK and are comparable to age-matched population singletons in terms of clinical phenotype and lifestyle characteristics (Verdi *et al.*, 2019). A total of 3477 twins were included in the analyses (1771 DZ twin individuals and 1706 MZ twin individuals used as controls).

#### TEDS UK study

The Twins Early Development Study (TEDS) is a longitudinal twin study that recruited over 16 000 twin-pairs born between 1994 and 1996 in England and Wales through national birth records (Rimfeld *et al.*, 2019). The sample consisted of a maximum of 6690 twins (4125 DZ twin individuals and 2565 MZ twin individuals used as controls).

#### Finnish Twin Cohort study

The Finnish Twin Cohort study comprises three longitudinal studies of twins born before 1958 (Kaprio *et al.*, 2019), twins born 1975–1979 (Kaidesoja *et al.*, 2019), and twins born 1983–1987 (Rose *et al.*, 2019). A total of 8188 twins were included in the analyses (6021 DZ twin individuals and 2167 MZ twin individuals used as controls).

#### Swedish Twin Registry

A sub-study of the Swedish Twin Registry denoted TwinGene was used to test single nucleotide polymorphism (SNP) associations between dizygotic (DZ) and monozygotic (MZ) twins. All included twins were born 1958 or earlier. A total of 6777 twins were included in the analysis (5373 DZ twin individuals and 1404 MZ twin individulas used as controls).

#### UKBiobank study

We analyzed data from UKB release 2. The UKB cohort contains data for 488 377 participants from across the UK, aged between 40 and 69 years, collected between 2006 and 2010. The closest available data on twinning is in the item: ‘Are you a twin, triplet or other multiple birth?’ (UKB questionnaire field ID 1777). The options were: ‘Yes’, ‘No’, ‘Do not know’, and ‘Prefer not to answer’. We focused on 8962 participants with Caucasian ancestry who reported being part of a multiple birth, and 409 591 controls. Detailed information on selection of twins can be found in Mbarek *et al.* (2019).

### Genotyping and quality control (QC)

Details on genotyping, imputation and QC are given in Supplementary Data File S1 and Supplementary Table S1. SNP data were imputed to either 1000 Genomes or the Haplotype Reference Consortium.

### GWAS and meta-analysis

A genome-wide association analysis was conducted in each study by logistic regression under an additive genetic model using Plink version 1.07. Analyses were adjusted for population structure using residues from the first ten principal components of genetic ancestry and sex (Logit (DZT vs controls) = SNP + sex + 10 PCs), except in the MoDZT-only analyses (Logit (MoDZT vs controls) = SNP + 10 PCs). Because the DZT GWAS data include family members, we used the –family option in the analysis, which takes the familial structure of the data into account using a sandwich estimator. For complete pairs, one twin per pair was randomly selected. Before meta-analysis, we removed SNPs with low minor allele frequencies (<0.01), low imputation quality (*r*^2^ < 0.8) and deviations from Hardy–Weinberg equilibrium (*P* < 10^−5^). A total of 8 553 805 variants met these criteria and were kept in the final results. Results from individual GWAS (MoDZT and DZT) were combined by the fixed-effects inverse variance method based on the regression β estimates and standard errors from each study implemented in METAL software (Willer *et al.*, 2010). Genotyped DZ twins whose mothers were not genotyped account for half of the genetic information of MoDZT. They were included in the meta-analysis taking account of the differential genetic information from mothers of twins and twin offspring (full details on the statistical calculation and simulation can be found in the Supplementary Data File S1). The genome-wide significance threshold was set at the level of *P* = 5.0 × 10^−8^. LocusZoom (http://locuszoom.org/) was used to provide regional visualization of the results. Full details of participants are in Table 1. Full details of the analysis plan can be found in the standard operating procedure circulated to the consortium study cohorts and inserted at the end of the Supplementary Data File S1 as well as the earlier papers of the Twinning Genetics Consortium (Mbarek *et al.*, 2016a,b, 2019).

### Follow-up analyses

#### Gene-based test and pathway analyses

We used two gene-based test methods. The first gene-based test is performed by MAGMA 1.08, followed by pathway analyses, and both are implemented in FUMA (Watanabe *et al.*, 2017), which employs multiple linear regression while Pascal computes the sum and maximum of chi-squared statistics to obtain gene-based *P*-values. The second gene-based and pathway-based test is VEGAS2 which performs permutation-based simulation (Mishra and Macgregor, 2015).

#### Zebrafish methods

Zebrafish (*Danio rerio*) husbandry and genotyping were based on the wild-type Tupfel long fin zebrafish strains which were raised according to standard protocols. All animal procedures were performed in accordance with French and European Union animal welfare guidelines with protocols approved by Sorbonne Université (APAFIS#21323-2019062416186982). Animal procedures were performed in accordance with French and European Union animal welfare guidelines and were approved by the Committee on ethics of animal experimentation (number 005).

The zebrafish *Ipo8* mutant line was genotyped as previously described (Zeigler *et al.*, 2021). Details on the quantification and hybridization can be found in the Supplementary Data File S1.

### Statistical analysis

Data were analyzed using GraphPad Prism software (GraphPad Software, San Diego, CA, USA) applying the non-parametric Mann–Whitney test.

## Results

### Meta-analysis, gene-based, and pathways analyses

**Figure 1.**
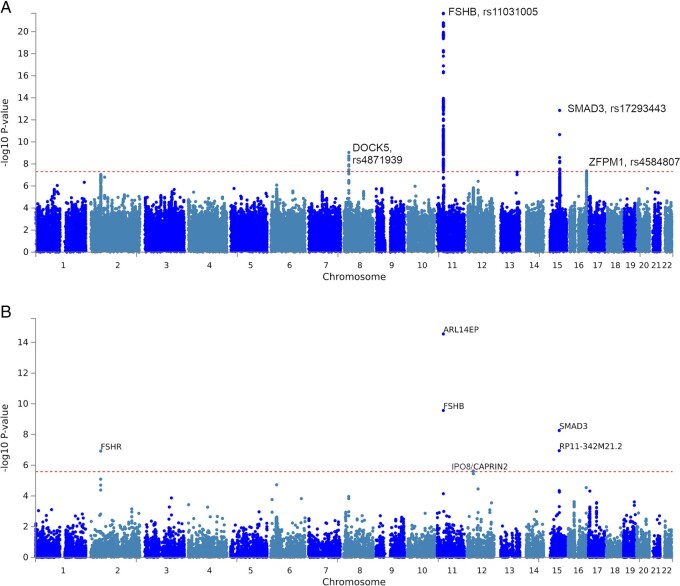
**Association results of the genome wide association study (GWAS) meta-analysis of dizygotic (DZ) twinning versus controls.** (**A**) Manhattan plot of single nucleotide polymorphisms (SNP) associations. The -log_10_ p value (*y*-axis) for the SNPs are plotted against their physical chromosomal position (*x*-axis). The dashed red line represents the genome-wide level of significance (*P* value< 5 × 10^−8^). The rs numbers and gene name indicate the chromosomal region attaining genome-wide significance. For plotting purposes, overlapping data points are not drawn for filtered SNPs with a *P*-value ≥1 × 10^−5^. (**B**) Manhattan plot of gene-based association results as computed by MAGMA. The -log_10_*P* value (*y*-axis) for the genes are plotted against their physical chromosomal position (*x*-axis). Input SNPs were mapped to 19171 protein coding genes. Genome-wide significance (red dashed line in the plot) was defined at *P* = 0.05/19171 = 2.61 × 10^−6^.

Results from the overall GWAMA identified four genome-wide significant (*P* < 5 × 10^−8^) loci including two novel regions: one near *DOCK5* (Dedicator of Cytokinesis 5) on chromosome 8 (rs4871939, OR = 1.08, *P* = 9.06 × 10^−10^) adjacent to *GNRH1* which is the likely causal gene (see below), and a second novel locus near *ZFPM1* (Zinc Finger Protein, FOG Family Member 1) on chromosome 16 (rs4584807, OR = 1.07, *P* = 4.4 × 10^−8^). Previous reported signals near *FSHB* and in *SMAD3* were replicated (Mbarek *et al.*, 2016b) (Fig. 1A and Supplementary Table S2). Gene-based tests in VEGAS2 (Mishra and Macgregor, 2015; Mishra and MacGregor, 2017) and MAGMA (v1.08) (de Leeuw *et al.*, 2015) showed highly concordant results (Fig. 1B, Table 2). At a genome-wide significance threshold of 2.6 × 10^−6^ classically used for gene-based tests, four genes were identified by both approaches: *FSHR, SMAD3* and *FSHB* and *ARL14EP* (adjacent to *FSHB*). In addition, VEGAS2 identified the microRNA *MIR5189* on chromosome 16, which is located within an intron of *ZFPM1* while MAGMA identified *CAPRIN2* adjacent to *IPO8* (Importin 8) on chromosome 12 which itself fell just short of significance by both methods; we provide functional evidence below that *IPO8* is the causal gene at this locus (Fig. 1B, Table 2, Supplementary Tables S3 and S4). Significant pathways identified from the VEGAS2 gene list included the Reactome pathway ‘Hormone ligand binding receptors’, ‘Rhythmic processes (including ovulation cycle and ovulation cycle processes)’, and the GO pathway 0042698, Ovulation Cycle, implicating the hypothalamic–pituitary–gonadotrophin axis and intra-ovarian signaling (Supplementary Table S5).

**Table 2.** Overall genome-wide association meta-analysis results and gene-based tests.

		P Gene-based test		Top SNP in gene						Top SNP outside gene					
Gene	Locus	MAGMA	VEGAS2	rsid	bp	a1/a2	RAF (eaf)	Beta (se)	*P*	rsid	pos	a1/a2	RAF	Beta (se)	*P*
ARL14EP	11p14.1	2.83 × 10^−15^	9.99 × 10^−7^	rs7929660	30339461	a/g	0.835	0.145 (0.015)	1.79 × 10^−21^	rs11031005	30226356	t/c	0.8662	0.1564 (0.0161)	2.17 × 10^−22^
FSHB	11p14.1	2.71 × 10^−10^	9.99 × 10^−7^	rs10835638	30252352	t/g	0.155	−0.136 (0.015)	1.67 × 10^−18^	rs74485684	30242287	t/c	0.8368	0.1453 (0.0153)	1.58 × 10^−21^
SMAD3	15q22.33	5.48 × 10^−9^	9.99 × 10^−7^	rs17293443	67437863	t/c	0.772	−0.098 (0.013)	1.39 × 10^−13^	rs12148306	68239614	a/t	0.4634	0.0651 (0.0112)	5.77 × 10^−9^
RP11-342M21.2	15q22.33	1.13 × 10^−7^	–	rs17293443	67437863	t/c	0.772	−0.098 (0.013)	1.39 × 10^−13^	rs16950687 (intron SMAD3)	67464013	a/g	0.7254	−0.074 (0.0124)	2.61 × 10^−9^
MIR5189	16q24.2	–	9.99 × 10^−7^	rs4584807	88528125	t/c	0.313	−0.068 (0.012)	4.39 × 10^−8^	rs12445547 (intron ZFPM1)	88518569	t/g	0.3047	−0.0599 (0.0125)	1.82 × 10^−6^
ZFPM1	16q24.2	2.88 × 10^−5^	7.99 × 10^−6^	rs4584807	88528125	t/c	0.313	−0.069 (0.012)	4.39 × 10^−8^	rs8045886	88508781	c/g	0.7344	0.0604 (0.0135)	7.84 × 10^−6^
DOCK5	8p21.2	1.49 × 10^−4^	2.86 × 10^−4^	rs4871939	25267103	a/g	0.248	−0.080 (0.013)	9.06 × 10^−10^	rs6185 (GNRH1 stop gained)	25280800	c/g	0.7475	0.0759 (0.0129)	4.34 × 10^−9^
GNRH1	8p21.2	1.08 × 10^−4^	6.19 × 10^−5^	rs4871939	25267103	a/g	0.248	−0.080 (0.013)	9.06 × 10^−10^	rs17053711 (intron KCTD9)	25311269	a/g	0.2665	−0.0724 (0.0127)	1.20 × 10^−8^
CAPRIN2	12p11.21	2.37 × 10^−6^	2.99 × 10^−6^	rs11051050	30897005	a/c	0.243	0.062 (0.013)	1.81 × 10^−6^	rs12824388	30919517	t/c	0.7336	−0.0608 (0.0126)	1.48 × 10^−6^
IPO8	12p11.21	3.53 × 10^−6^	2.99 × 10^−6^	rs10771757	30831037	t/c	0.242	0.061 (0.013)	2.47 × 10^−6^	rs12830910 (intron CAPRIN2)	30890234	t/c	0.7449	−0.0605 (0.0128)	2.19 × 10^−6^
FSHR	2p16.3	1.20 × 10^−7^	1.99 × 10^−6^	rs12473870	49292341	a/g	0.405	0.060 (0.011)	1.15 × 10^−7^	rs10196478	49454765	t/c	0.8828	−0.0927 (0.0173)	9.08 × 10^−8^
STON1-GTF2A1L	2p16.3	8.08 × 10^−6^	1.09 × 10^−5^	rs13014919	48882878	a/g	0.718	−0.049 (0.012)	8.09 × 10^−5^	rs12619514	49033183	t/g	0.4949	0.0439 (0.111)	8.02 × 10^−5^
LHCGR	2p16.3	1.98 × 10^−5^	1.04 × 10^−4^	rs34790224	48912007	t/c	0.218	0.052 (0.013)	1.08 × 10^−4^	rs13014919 (intron STON1-GTF2A1L)	48882878	a/g	0.7184	−0.0487 (0.0124)	8.10 × 10^−5^
SHBG	17p13.1	5.30 × 10^−4^	4.23 × 10^−4^	rs1799941	7533423	a/g	0.259	0.056 (0.013)	8.78 × 10^−6^	rs12452603	7504977	t/c	0.7210	−0.0530 (0.0124)	1.92 × 10^−5^
HLA-G	6p22.1	1.88 × 10^−5^	1.08 × 10^−5^	rs1611144	29799354	a/g	0.538	−0.054 (0.011)	1.86 × 10^−6^	rs2523764	29817617	a/g	0.6726	−0.0589 (0.012)	8.32 × 10^−7^

Results of gene-based tests for top loci in the genome wide association study (GWAS) meta-analysis for DZ twinning, with *P* values for both MAGMA1.08 and VEGAS2. For VEGAS2, the gene-based P-value is an empirical one based on simulation; up to 1 million simulation replicates are generated, which places a lower bound on *P* values of 9.99 × 10^−7^. SNP: single nucleotide polymorphism.

**Figure 2.**
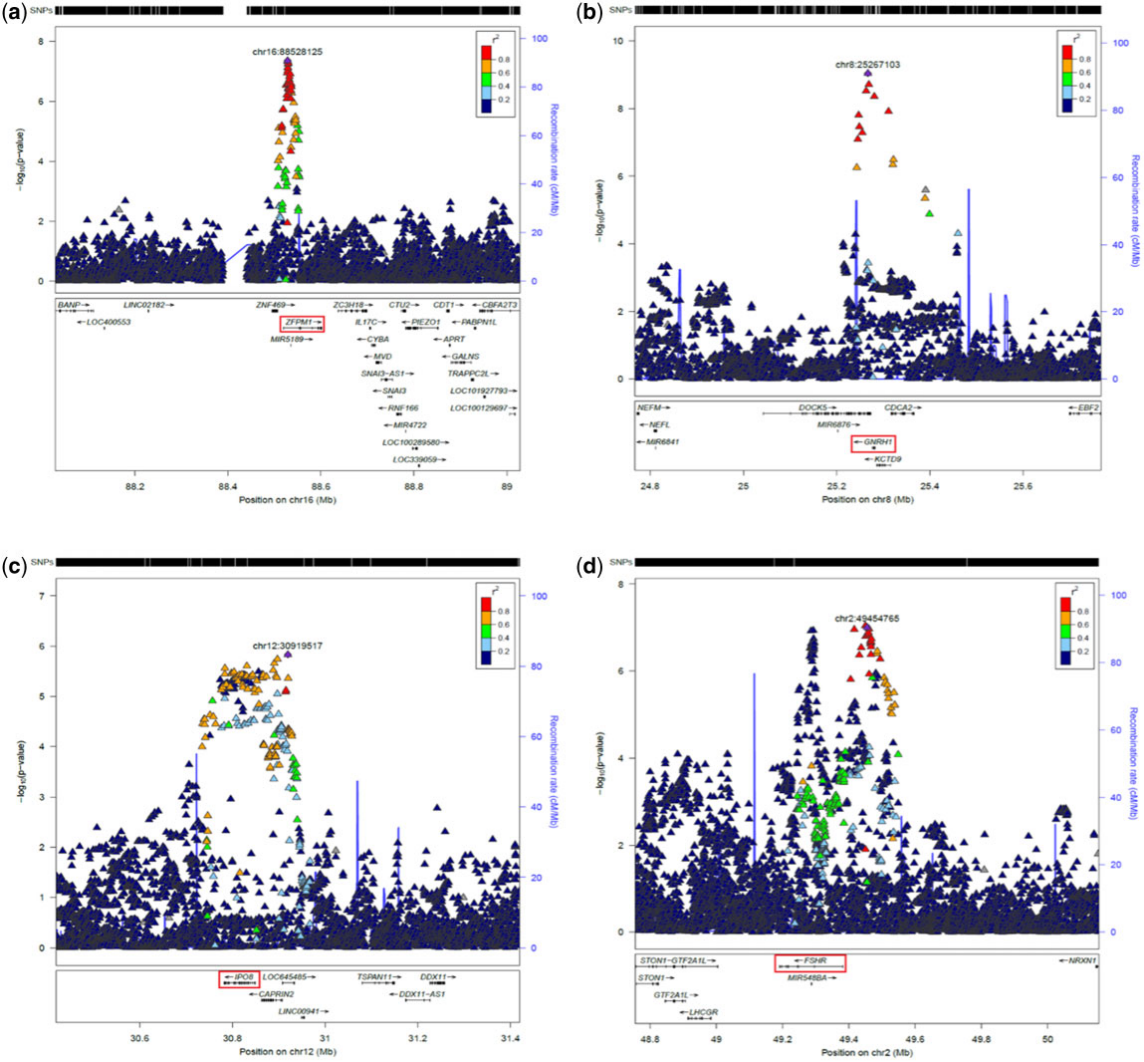
**Regional association plots for genome wide association study (GWAS) meta-analysis, for newly-reported associations.** Regional association plots for the four new regions reported in Table 2. The reference single nucleotide polymorphism (SNP) is the most highly associated one reported. The suggested gene in the text is highlighted in red. Loci are: (**a**) 16q24.2; (**b**) 8p21.2; (**c**) 12p11.21; (**d**) 2p16.3. Approximate conditional analysis in GCTA on lead SNPs (not shown) shows only one significant association in each case. Plots were prepared using a stand-alone copy of LocusZoom 1.3 (http://locuszoom.org/) for the 1000G Release 3 European LD reference, and hg19/Build 37.

Regional Association Plots (RAPs, Fig. 2a–d) show the four significant associations in detail (SNP-based: 16p24.2/*ZFPM1*; 8p21.2/*DOCK5*, *GNRH1*; Gene-based: 12p11.21/*CAPRIN2, IPO8*; and 2p16.3/*FSHR*). Conditional analyses (not shown) indicate that these each contains only one significant association signal. RAPs for the two replicated loci (11p14.1/*ARL14EP, FSHB*, and 15q22.33/*SMAD3, RP11-342M21.2*) as well as the two regions which were below formal significance level but were still functionally relevant (17p13.1/*SHBG* and 6p22.1/*HLA-G*) are shown in Supplementary Fig. S1a–d.

### Heritability and polygenic risk scores

Based on genome-wide complex trait Bayesian analyses (GCTB) software (Zeng *et al.*, 2018), the common SNP-based heritability of DZ twinning is estimated at ∼0.5% on the observed risk scale and ∼2.4% on the liability scale assuming a prevalence of DZ twinning of 1%. This SNP-based heritability estimate is broadly compatible with the accuracy of a polygenic risk score (PRS) for DZ twinning quantified in the Netherlands Twin Register (NTR) (1.3%, see below). These estimates are lower than those derived from Bulmer (Bulmer, 1970) and Lewis (Lewis *et al.*, 1996). Bulmer (in Table 6.6) reports sister-sister recurrence data that are compatible with a tetrachoric correlation in liability of 0.14, i.e. a heritability of 28%, while Lewis et al. estimated a heritability of 0–26% from recurrence data of DZ offspring in MZ twin mothers (Lewis *et al.*, 1996). The heritability estimates came from seven large multigenerational pedigrees from West Africa, fin-de-siècle French Jewish populations, Canadians and the French royal family, in which the twin births recorded were 8–20%. The estimates are remarkably consistent across time (8–19 generations) and ethnicities (Duffy and Martin, 2022), and probably can be up-weighted by as much as a third as zygosity was not known.

Leaving out the NTR sample, we re-ran the meta-analysis to obtain the summary statistics from the remaining discovery samples and used these to calculate PRS with LDPRED in a target sample of 1186 cases and 3377 controls (Supplementary Fig. S2). The highest correlation between PRS and case status was for a PRS based on the LDPRED top 1000th (*P* = 0.001) fraction of all summary statistics SNPs, resulting in a Spearman correlation 0.095, *P* < 0.001 and explaining 1.3% of variance on the liability scale after correction for 10 genotypic principal components and genotype platforms. The results show an increasing risk of giving birth to DZ twins with the top decile of PRS having 2.5 times the risk of giving birth to DZ twins compared with the bottom decile (Supplementary Fig. S3). We also calculated PRS for two special out-of-sample studies of DZT-dense pedigrees in Belgium (21 cases) and Utah (10 cases). We did not see elevated PRS in these samples relative to Australian controls (Supplementary Fig. S4) but we did see enrichment of top SNPs (particularly *GNRH1*) in the Belgian pedigrees (Supplementary Table S6).

### Genetic correlations

**Figure 3.**
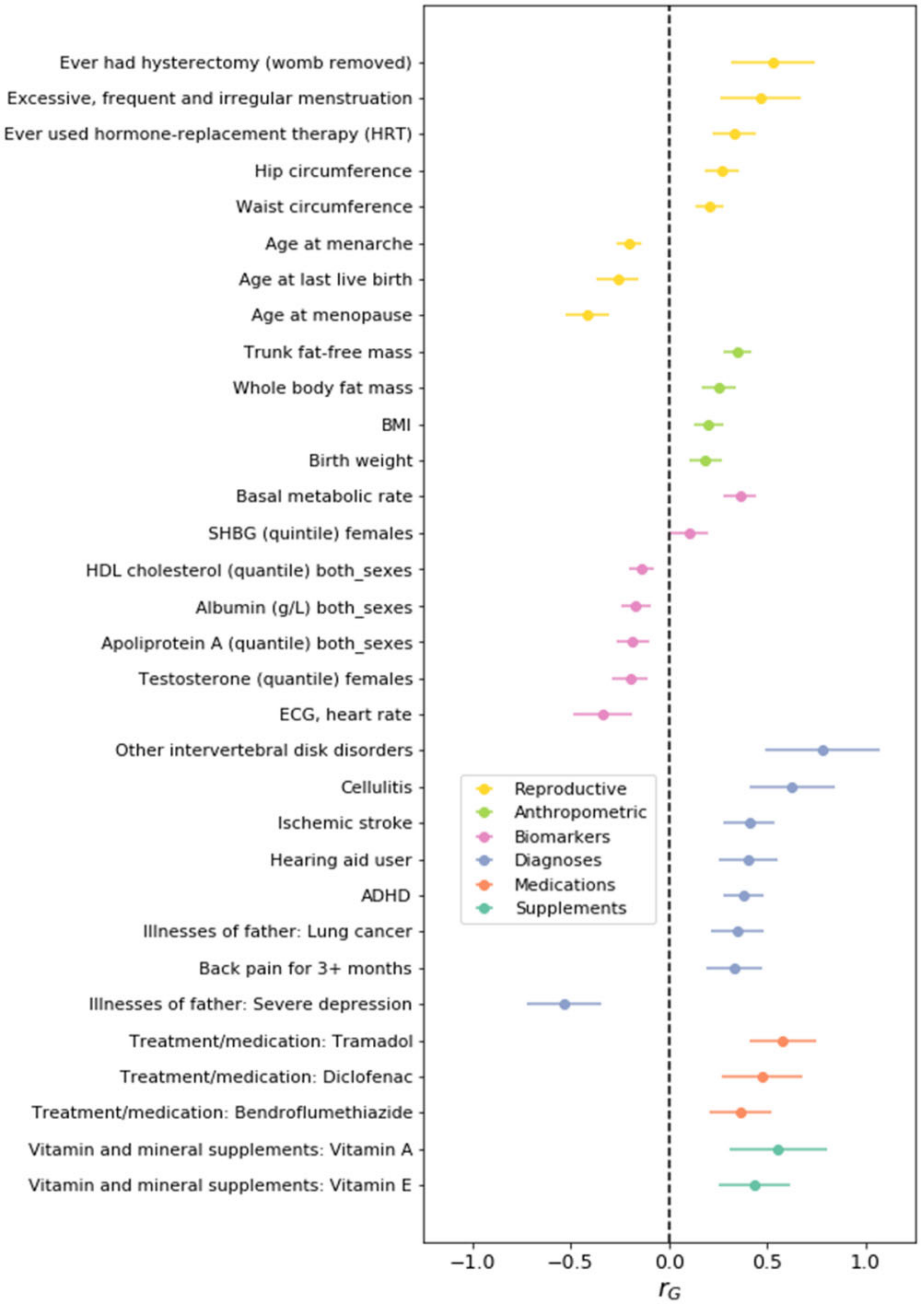
**Genetic relationships between the genome wide association study (GWAS) meta-analysis results of dizygotic (DZ) twinning and 28 other traits**. Single nucleotide polymorphism (SNP)-based genetic correlations (r_g_) were estimated with linkage disequilibrium (LD) score regression. The bars represent the standard error of r_g_. The genetic correlation estimates are colour-coded by trait category. BMI, body mass index; SHBG, sex hormone binding globulin; HDL, high-density lipoprotein; ECG, electrocardiogram; ADHD, attention deficit hyperactivity disorder.

Based on large GWAMAs for selected health-related and anthropometric traits in the Complex-Traits Genetics Virtual Lab (CTG-VL) (Bulik-Sullivan *et al.*, 2015; Haworth *et al.*, 2021) (Supplementary Table S7), LDHub (Zheng *et al.*, 2017) (Supplementary Table S8), and for sex hormones (Ruth *et al.*, 2020) in (Supplementary Table S9), we found significant genetic correlations with other measures of female reproduction, e.g. earlier menarche and greater number of children and larger body size (Fig. 3). Mothers of DZ twins tend to have earlier menopause (Martin *et al.*, 1997; Gosden *et al.*, 2007), possibly reflecting earlier depletion of oocytes. Also, mothers of DZ twins smoke more often (Olsen *et al.*, 1988; Hoekstra *et al.*, 2010), a finding that has been attributed to the anti-estrogen effect of smoking. Consistent with this, we observe a positive genetic correlation for DZ twinning with lung cancer and other cancers associated with smoking and with smoking itself (Supplementary Table S10).

### Phenome-wide association study (PheWAS)

PheWAS based on the GWAMA, associated SNPs predisposing to DZT with reduced risk of both ovarian dysfunction and polycystic ovaries (Supplementary Fig. S5, Supplementary Table S11). Since both conditions negatively correlate with fertility, we hypothesize that any shared genetic effects with DZT are driven through fertility in opposing directions.

### *In silico* functional analysis

We sought evidence for the biological mechanism underlying the lead SNPs in each of our top regions. The lead SNP on chromosome 8 (rs4871939) is located in an intron of *DOCK5*, immediately proximal to hypothalamic gonadotropin releasing hormone 1 (*GNRH1*) gene and is in high linkage disequilibrium (LD) (*r*^2^ > 0.94) with rs6185, a coding sequence variant in *GNRH1* and two other SNPs associated, from previous GWAS, with age at menopause (Horikoshi *et al.*, 2018), length of the menstrual cycle (Laisk *et al.*, 2018), total testosterone levels (Ruth *et al.*, 2020), and heel bone mineral density (Morris *et al.*, 2019). These four SNPs form a single haplotype with two alleles and the common allele (frequency 0.75) is associated with increased DZ twinning. GNRH is implicated in ovarian function and DZ twinning through the role of GNRH pulse frequency from the hypothalamus regulating FSH and LH synthesis and release from the pituitary gland (Mbarek *et al.*, 2016b). Higher pulse frequency of FSH is reported in mothers of DZ twins (Lambalk *et al.*, 1998). However, roles for other genes in the region cannot be ruled out. *DOCK5* is differentially expressed across the menstrual cycle (*P* < 2 × 10^−16^) with high expression in the secretory phase, suggesting it is responsive to sex hormone changes across the cycle (Mortlock *et al.*, 2020).

The lead SNP for the novel locus, rs4584807 on chromosome 16, is located in a regulatory region in the first intron of *ZFPM1*, which encodes a transcription regulator that plays an essential role in erythroid and megakaryocytic cell differentiation. ZFPM1 is an essential cofactor which acts by forming heterodimers with transcription factors of the GATA family, *GATA1*, *GATA2*, and *GATA3.* This signal is also in high LD (*r*^2^ > 0.93) with SNPs in a region associated with age at menopause (Kichaev *et al.*, 2019), sex hormone binding globulin (Ruth *et al.*, 2020), and FSH concentrations (Chen *et al.*, 2013), suggesting a direct effect of the causal SNP(s) through altered FSH concentrations.

The lead SNP on chromosome 11p14 (rs11031005) is located upstream of *FSHB*, the gene encoding the beta polypeptide of FSH. The allele associated with increased twinning was also associated with increased FSH and decreased luteinizing (LH) concentrations, in agreement with previous results (Ruth *et al.*, 2016).

### Functional links with hormone levels

**Figure 4.**
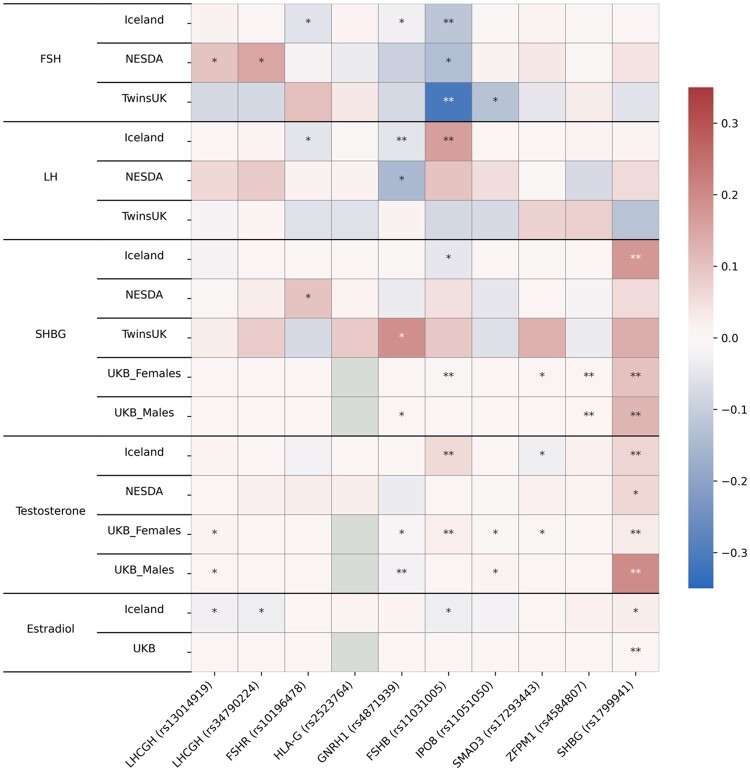
**Association of top dizygotic (DZ) twinning single nucleotide polymorphisms (SNPs) with *in vivo* reproductive hormone data from four independent studies.** Heat map showing the beta value of the association of each DZ twinning SNP and corresponding hormone data supplied by the indicated study. SNPs with a positive effect (positive beta) are shown in red, SNPs with a negative effect (negative beta) are shown in blue. Darker shading indicates beta values of larger magnitude. A single asterisk (*) indicates nominal significance at alpha = 0.05, and the double asterisk (**) indicates significance at the alpha corrected for multiple testing, i.e. alpha = 0.05/(10 × 17). Cells in light gray represent missing/unavailable data. Additional details, including study sample sizes and hormone measurement specifications, can be found in Supplementary Table S12. LHCGH, luteinizing hormone/choriogonadotropin hormone; FSHR, follicle stimulating hormone receptor; HLA-G, human leukocyte antigen G; GNRH1, gonadotropin releasing hormone 1; FSHB, follicle stimulating hormone subunit beta; IPO8, importin 8; SMAD3, mothers against decapentaplegic homolog 3; ZFPM1, zinc finger protein; FOG, Family Member 1; SHBG, sex hormone binding globulin; FSH, follicle stimulating hormone; LH, luteinizing hormone; Test, total testosterone; Est, estradiol. Iceland (deCODE), NESDA, TwinsUK and UKB are the four studies.

To identify functional links with hormone levels, we performed a look-up of the most likely SNPs in our top regions on in vivo levels of five reproductive hormones (FSH, LH, sex hormone binding globulin (SHBG), testosterone (T), and estradiol (E)); unfortunately, assays of inhibin A and B levels, which are strongly involved in ovulation, were unavailable. Look-ups were done in four different cohorts (NESDA (Bot *et al.*, 2015), deCODE (Gudbjartsson *et al.*, 2015), UKB (Ruth *et al.*, 2020), and TwinsUK (Ruth *et al.*, 2016)) which assessed overlapping sets of hormones, but sometimes by different techniques (Fig. 4, Supplementary Table S12), or different fractions (e.g. total, bioavailable (free/unbound) testosterone). Multiple associations were seen, most notably of the top *FSHB* SNP with *in vivo* levels of FSH, LH, SHBG, and T. The top *FSHR* SNP also associated with FSH, LH and SHBG but not T. The *GNRH1* SNP was significantly associated with decreased in vivo levels of FSH, LH and T and increased levels of SHBG. Levels of SHBG were also associated with the top SNP in *ZFPM1* and with rs1799941 on chromosome 17 in the *SHBG* gene. This SNP falls just below the threshold for GWAS significance for DZ twinning, but strongly affects *in vivo* levels of SHBG, T, and E and has previously been shown to increase SHBG levels, which in turn lowers estradiol and increases FSH (Ruth *et al.*, 2020). Most results were seen in only some of the studies, with SHBG and FSH levels showing the most consistent findings. FSH is the best-established link between sex hormone levels and DZ twinning, and essentially, higher (free) estradiol should suppress FSH and reduce DZ twinning. Thus, alleles which raise SHBG should lower free estradiol and increase FSH, and hence DZ twinning (Supplementary Table S12).

### Expression quantitative trait (eQTL) analysis

Genomic regions associated with twinning overlap with eQTL signals for 11 genes in multiple tissues in GTEx and 8 genes expressed in blood (eQTLGen). We conducted SMR (Summary-based Mendelian Randomization) (Zhu *et al.*, 2016) analysis to evaluate whether the same causal variants influenced DZ twinning and gene expression. *ARL14EP* is located next to *FSHB* on chromosome 11. It is widely expressed with relatively high expression in ovaries and testes (GTEx Consortium *et al.*, 2017). One SNP rs4071559 in high LD with our lead SNP rs11031005 (*r*^2^ = 0.82) is an eQTL for *ARL14EP* in the testis (Supplementary Fig. S6, Supplementary Table S13). This association was significant in the SMR test, and also passed the heterogeneity in dependent instruments (HEIDI) (Zhu *et al.*, 2016) test. This suggests that altered expression of *ARL14EP* may be associated with variation in twinning, supporting the transcriptome-wide association study (TWAS) results below.

The gene-based tests implicated both the FSH receptor *FSHR* and *CAPRIN2*. *CAPRIN2* is co-located with Importin 8 (*IPO8*) on chromosome 12. Both genes are expressed in multiple tissues with significant expression in the ovary and uterus. The variant rs10843810 on chromosome 12 associated with DZ twinning is an eQTL for *IPO8* in endometrium; it was significant in the SMR test and passed the HEIDI test, suggesting a possible causal relation between the variant associated with DZ twinning and expression of *IPO8* (Supplementary Table S14). *IPO8* is required for TGFβ-activated SMAD2/3 to translocate into the nucleus (Xu *et al.*, 2007; Yao *et al.*, 2008) and is therefore a likely candidate to attempt functional validation in zebrafish (see below).

The lead SNP on chromosome 16 near *ZFPM1*, rs4584807, is in high LD with nearby SNPs associated with age at menopause (Mbarek *et al.*, 2016b, 2019), sex hormone binding globulin (Ruth *et al.*, 2020) and FSH concentrations (Mbarek *et al.*, 2016b; Ruth *et al.*, 2016). *ZFPM1* is also significantly differentially expressed across the menstrual cycle in the endometrium (*P* = 4.81 × 10^−5^), with highest expression in the menstrual phase of the cycle (Mbarek *et al.*, 2016a,b). ZFPM1 and other FOG proteins act as repressors on multiple gonadal promoters in the testis, modulating the expression of GATA-dependent genes including effects on steroidogenesis and P450 aromatase (Robert *et al.*, 2002), and may influence gonadotrophin concentrations through steroid hormone feedback.

### Transcriptome-wide association study (TWAS)

We performed a TWAS in MetaXcan based on Joint Tissue Imputations (JTI) models in eight tissues involved in reproduction or hormonal regulation: breast, hypothalamus, ovary, pituitary gland, testis, uterus, vagina, and whole blood tissue (Gamazon *et al.*, 2015) (Supplementary Table S15). In total, 22 genes were significant at a tissue-specific significance level (ranging from 7.04 × 10^−6^ to 3.00 × 10^−6^ across tissues, depending on the number of genes per tissue) while eight genes survived a tissue-wide significance level (∝ = 6.74 × 10^−7^). These eight included four genes that were also identified in the GWAS analysis: *ARL14EP*, *CAPRIN2*, *ZFPM1*, and *SMAD3* (Supplementary Table S16, Supplementary Fig. S7) and *IPO8*, which was almost significant. The *IPO8* expression was reduced in cases, compared to controls, in uterus, testis, hypothalamus, breast, and whole blood, with effect sizes ranging from −0.16 to −0.10. In these tissues, the association was significant when correcting for the number of tests per tissue, although none of the associations were significant when controlling for the total number of tests across tissues. The TWAS identified *ARL14EP* as the top hit in nearly all tissues and implies decreased levels of genetically regulated *ARL14EP* expression in MODZT and DZ twins, compared to controls (Supplementary Fig. S7). This analysis provides additional support for the involvement of *ARL14EP* in DZ twinning, although its likely target is the neighboring *FSHB* gene. In addition, we identified four other genes via TWAS, *MPPED2-AS*, *GOLGA8T*, *PCBP2*, and *FAM66D*, although these are not yet supported by other analyses.

### Global DZ twinning rates

A remarkable feature of spontaneous DZ twinning rates is that they vary widely between major ancestry groups, with a 10-fold difference between individuals with West African up to 20/1000 births, with 42/1000 births in Western Nigeria (Nylander, 1969) and East Asian (2/1000) and European ancestries (8/1000). These rates are from the 1960s, i.e. before the introduction of ART, and were observed in relatively young women (Bulmer, 1970; Pison and D’Addato, 2006). Frequencies of our top SNPs are illustrated in Supplementary Fig. S8 for the major extant ancestry groups along with available data from archaic DNA and certain primates (Haak *et al.*, 2005). Some frequencies show a gradient in the expected direction with the risk allele being most frequent in African populations and least frequent in Asians, with Europeans in between, but the gradients of others go in the opposite direction or are inconsistent. We observed that the derived allele is associated with twinning for *IPO8*, *SMAD3*, and *GNRH1*, but not for *FSHB*, where the derived allele is associated with decreased DZT. Of the four genes, only *IPO8* shows an ancestry group trend consistent with DZT frequencies. For *GNRH1*, the derived allele is the major allele, and seems to be present in Denisovans, as well as in Pleistocene anatomically modern humans, although the low LD limits inference. For *ZFPM1* and *FSHR*, we have no ancestral data so can only compare the extant population frequencies of the DZT predisposing alleles. Our top SNPs show wide geographic variation in frequencies (Supplementary Fig. S9) and, even though they collectively account for only 0.9% variance in European ancestry individuals (against a total SNP heritability h^2^s ∼2.4% and multifactorial threshold h^2^ ∼15%), we combined them based on population specific allele frequencies with the effect sizes from our GWAMA in Europeans to predict the twinning rate in diverse world populations.

**Figure 5.**
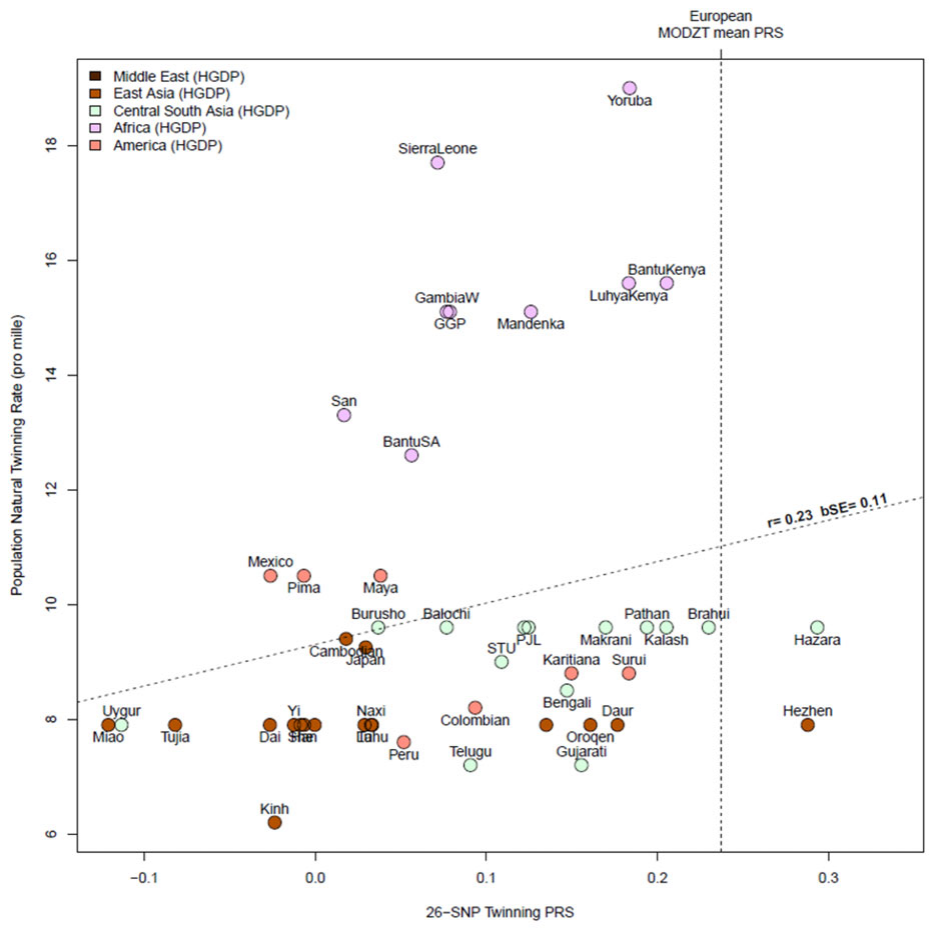
**The correlation between population twinning rates from 53 populations and predicted dizygotic twin (DZT) polygenic risk score (PRS) for each population based on European effect sizes of top single nucleotide polymorphisms (SNPs) from Table 2.** The twinning rate data are derived from Smits & Monden Demographic and Health Surveys (Smits and Monden, 2011). The HGDP group twinning rates are taken as those of the country from which they derive (e.g. Mandinka from Senegal); we have added twinning rate data for UK and Japan from Imaizumi (Imaizumi, 2003), other European twinning rates from Heino *et al.* (2016) and rates for Australia from the Australian Bureau of Statistics, 2010. The SNPs and their effect sizes are the 26 (unique) top associated SNPs from our genome wide association meta-analysis (GWAMA) listed in Table 2, excluding (for technical reasons) rs17293443, rs16050687, and rs17293443. Allele frequencies were obtained from the Human Genome Diversity Project and the 1000 Genomes Projects. We calculated the Pearson correlation and a bootstrap standard error (R boot package, 1000 replicates) using country-level rate and mean PRS for 53 populations. The vertical dotted line is the mean PRS for all mothers of DZ twins in the current study. The oblique line shows the regression line of observed twinning rate on the predicted DZT PRS for each population.

We extracted estimates of country-level natural population (total) twinning rate (per 1000 confinements) from three publications (Imaizumi, 2003; Smits and Monden, 2011; Heino *et al.*, 2016), and tested the correlation with a DZ twinning PRS, based on the 26 SNPs listed in Table 2 in samples from the present study, the Human Genome Diversity Project and the 1000 Genomes Project. We calculated the Pearson correlation and a bootstrap standard error (R boot package, 1000 replicates) from country-level rate and the mean PRS for 47 non-European populations; to avoid potential bias, European populations were not included in this comparison. The analyses are based on the total twinning rate, which is available for all populations, rather than the estimated DZ twinning rate, which is only available for some (Fig. 5). Thus, this will include MZ twins (about 4/1000 in all populations) and is unadjusted for maternal age and the use of ART, as the information for these is only available for some populations. Despite this uncertainty, the overall correlation in non-European populations between mean country 26-SNP DZ PRS and total twinning rate was *r* = 0.23 with a bootstrap SE = 0.11 (randomization *P* value (one-tailed) = 0.058, 10 000 replicates), suggesting that the variants that we have identified account for some, but not all, of the differences in global twinning rates.

### Evidence of selection against DZT

We used two approaches to test whether DZT has been undergoing natural selection (Supplementary File S1). We found genome-wide selection signals that suggest long-term selection against the tendency to DZ twinning through much of human history, consistent with a reduction of fecundity in the mammalian lineage and the trend toward smaller litter size (Supplementary Fig. S10).

### Zebrafish functional validation

We next sought to functionally validate rs10843810 (*P* = 4.34 × 10^−06^) near the *IPO8* gene, i.e., a potential new hit in our GWAS meta-analysis. This signal indicates that increased expression of the transcript of *IPO8* in mothers is associated with higher incidence of spontaneous DZ twins. While the hit did not reach a stringent genome-wide significance set at ∝ <5 × 10^−8^, its encoded protein IMPORTIN8 is a well-known and direct interactor of SMAD3 (Yao *et al.*, 2008), the second-highest GWAS hit for DZ twinning. SMAD3, which normally resides in the cytoplasm, becomes phosphorylated at its C-terminus upon TGFβ stimulation, after which it must be translocated in the nucleus to activate target genes (Feng and Derynck, 2005). The shuttling of pSMAD3 between cellular compartments is mediated in part by Importin8, with which it forms a molecular complex. In this context, optimal SMAD3 signaling depends on the availability of Importin8, and inhibition of either component is expected to decrease female fecundity. To test this hypothesis, zebrafish carrying an *ipo8* knockout allele (Ziegler *et al.*, 2021) were evaluated for signs of infertility.

**Figure 6.**
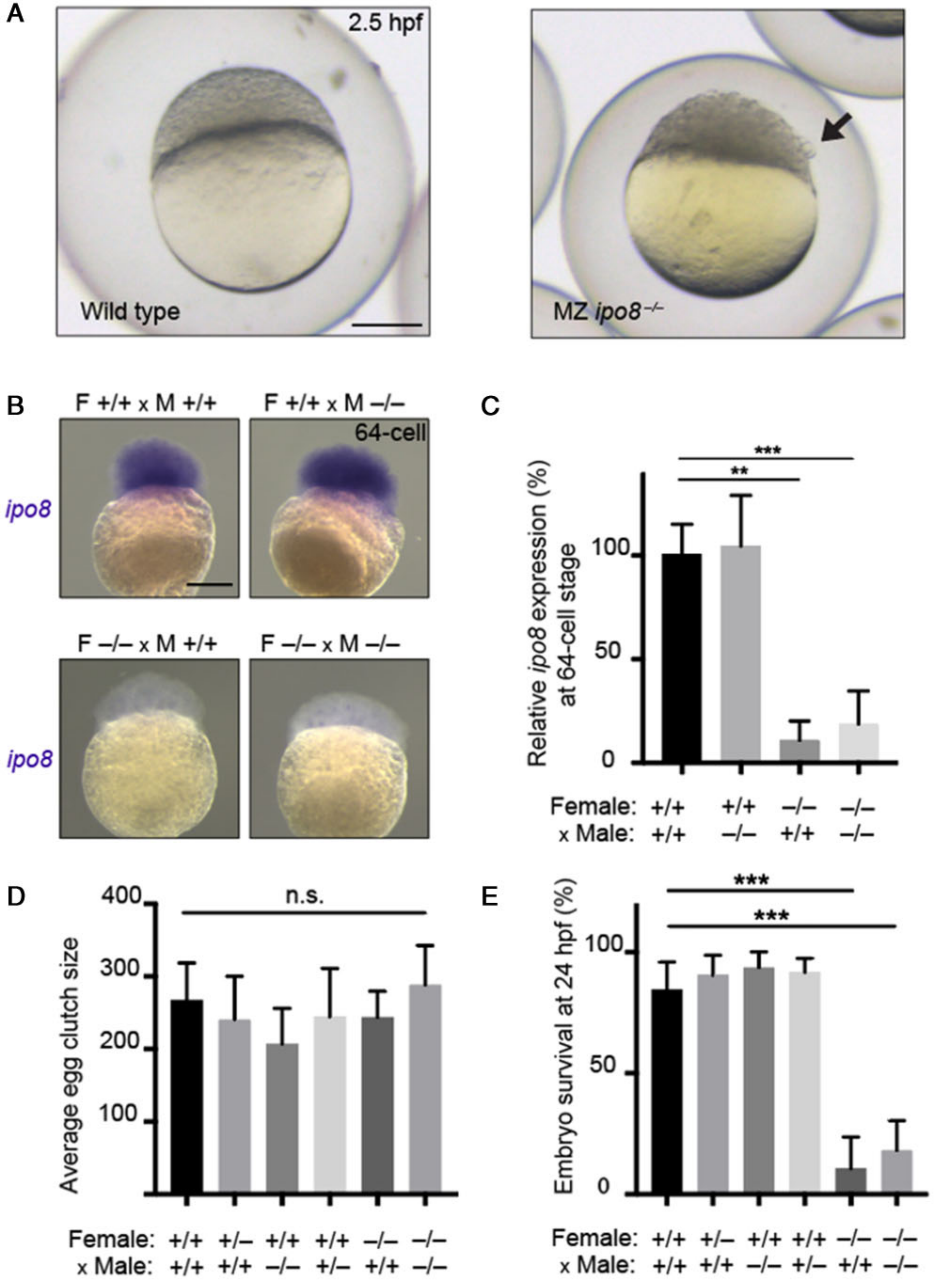
***ipo8* deficiency in zebrafish compromises maternal, but not paternal, fertility.** (**A**) Representative wild type (left) and maternal zygotic *ipo8* knockout (right) zebrafish embryo at 2.5 hpf. Embryos devoid of *ipo8* show a less compact dome with loosened blastomeres, as indicated by the black arrows. Scale bar 250 µm. (**B**) At the 64-cell stage, prior the onset of zygotic transcription, endogenous *ipo8* mRNA, revealed by whole-mount in situ hybridization, is contributed through maternal deposition in the egg and is absent in embryos derived from homozygous *ipo8* knockout females. Scale bar 250 µm. (**C**) Endogenous *ipo8* mRNA present in the 64-cell stage, measured by QPCR, originates from the maternal, but not paternal, contribution. (**D**) The size of clutches laid by wildtype, heterozygous or homozygous *ipo8* knockout mothers is not statistically different. Paternal *ipo8* genotype has no effect on egg clutch size. N > 6 for each cross. (**E**) Survival of embryos at 24 h post fertilization is solely dependent on maternal, but not paternal, presence of wild type *ipo8*. All error bars show 95% confidence interval. ∗∗*P* = <0.01, ∗∗∗*P* = <0.001; Mann–Whitney test.

Maternal zygotic knockout *ipo8* embryos born to parents entirely devoid of *ipo8* were indistinguishable from wild-type fish at fertilization. However, by the dome-stage (2.5 h post fertilization), they showed loose blastomeres detaching from the animal pole (Fig. 6A) resulting in early death. This phenotype was traced to maternal deposition of *ipo8* mRNA in the oocyte, because 64-cell embryos born to knockout (KO) fathers, but not KO mothers, had similar levels of *ipo8* as wild-type embryos. This was documented both by whole mount in situ hybridization and quantified by QPCR (Fig. 6B and C). This finding is consistent with the fact that in zebrafish zygotic genome transcription is initiated at the 64-cell stage, and prior to that point, the embryo relies on the maternally deposited mRNA available in the oocyte (Solnica-Krezel, 2020). We counted the size of clutches laid by wildtype, heterozygous, and homozygous *ipo8* KO mothers, but found no statistical differences, which suggests that the quantity of eggs is not dependent on *ipo8* levels during oogenesis (Fig. 6D). In contrast, we found that the quality of these embryos was severely compromised if laid by complete *ipo8* KO mothers, resulting in more than 75% fatality by one day post fertilization (Fig. 6E). These results indicate that maternal *ipo8* is essential to guarantee the quality and viability of embryos. If this can be transposed to fecundity in women, the role of IPO8 may be to ensure the viability of ovulated eggs, rather than to control the number of eggs released during each menstrual cycle. This would be consistent with the earlier discussion on how DZT etiology relies not only on multiple ovulation but also on overcoming the hazards of multiple pregnancy.

## Discussion

Our results confirm a role for common genetic risk factors which influence variation in DZ twinning in women and which appear to act mainly through a common pathway influencing FSH levels and/or sensitivity to FSH.

DZ twinning shows age-specific changes in the twinning rate (Hoekstra *et al.*, 2008; Otta *et al.*, 2016), which are thought to result from reduced hormonal feedback as the ovarian follicle pool decreases. This leads to higher FSH concentrations at the time of follicle selection, and more frequent selection of two dominant follicles (Lambalk *et al.*, 1998; Beemsterboer *et al.*, 2006). This is not sustained as the ovarian follicle pool diminishes further and DZ twinning rates decline with the approach of menopause. Therefore, genetic risk factors and age specific changes in DZ twinning rates likely act through effects on key genes (*GNRH1*, *FSHB*, *SMAD3*) in the hypothalamic–pituitary–ovarian axis and a fourth locus near *ZFPM1* that may modify FSH concentrations. In addition, reduced hormonal feedback with age modulates FSH signaling at the time of ovarian follicle selection (Lambalk *et al.*, 1998; Matzuk *et al.*, 2002; Fabre *et al.*, 2006; Kirkpatrick and Morris, 2015; Robker *et al.*, 2018). Increasing FSH concentrations, lowering the FSH threshold at the time of follicle selection, or increased sensitivity to FSH can increase the selection window allowing two follicles to be selected, leading to a twin pregnancy (Lambalk *et al.*, 1998; Vinet *et al.*, 2012).

Our results replicate previous findings on chromosome 11 near *FSHB* and chromosome 15 near *SMAD3*. The lead SNP, rs11031005 on chromosome 11 close to *FSHB*, is associated with DZ twinning and gonadotrophin concentrations, and is in high LD with four SNPs located in the 37 kb region upstream of the promoter of *FSHB*. FSH plays a critical role in regulating antral follicle growth, recruiting the dominant follicles(s), and regulating the ovulation rate (Lambalk *et al.*, 1998; Stamatiades and Kaiser, 2018). The four SNPs are on a common haplotype (frequency 0.82), which is associated with many reproductive traits and diseases, including earlier age at menarche and menopause (Day *et al.*, 2015, 2017), shorter menstrual cycles, increased risk of endometriosis (Sapkota *et al.*, 2017), and decreased risk of polycystic ovarian syndrome (Day *et al.*, 2018). Both rs11031005 and rs11031006 are correlated with a promoter polymorphism (c.-211G>T, rs10835638; *r*^2^ = 0.67) which is upstream of the transcription start site of *FSHB* and is reported to regulate *FSHB* gene transcription (Mbarek *et al.*, 2016b; Conforti *et al.*, 2019). This was also recently confirmed in luciferase assay experiments that identify a novel upstream regulator of FSHB transcription (Bohaczuk et al., 2021). Compared to women with the GG genotype, women with the FSH-decreasing GT genotype at rs10835638 more frequently have a poor response to ovarian stimulation (47.4% vs 26.5%, *P* = 0.010) (Trevisan *et al.*, 2019). *FSHB* is the likely target gene for variation on chromosome 11 influencing DZ twinning. However, a role for *ARL14EP* cannot be ruled out given evidence for a correlated SNP influencing expression of *ARL14EP*.

The lead SNP on chromosome 15 maps to the first intron of *SMAD3*, which was also significantly associated with DZ twinning in the gene-based test. *SMAD3* is expressed substantially in the human ovary, where it promotes granulosa cell proliferation and steroidogenesis (Mbarek *et al.*, 2016b). A major gene which increases the rate of ovulation and twinning in cattle maps to the equivalent genomic region on bovine chromosome 10 (Kirkpatrick and Morris, 2015), which contains both *SMAD3* and its paralogue *SMAD6*. An expression analysis in granulosa cells from cattle which are carriers or non-carriers of the gene demonstrated a 6-fold increase in expression of *SMAD6* in the gene carriers, suggesting that *SMAD6* is the target gene in cattle (Kamalludin *et al.*, 2018). However, analysis of chromatin states in multiple human tissues, including blood and ovary, shows that SNPs associated with DZ twinning are located in an enhancer with chromatin interactions to the promoter region of *SMAD3*, suggesting that *SMAD3* is the target gene for effects on human DZ twinning. Further studies are required to determine the target gene(s) in this region.

There are striking differences in global DZ twinning rates. We cannot discard the possibility that environmental influences differ between populations. The influence of eating certain yams and other traditional foods that are high in estrogenic compounds has been postulated to account for the high DZT rate in West Africa (Omonkhua *et al.*, 2020). However, the fact that DZT rates are also high in African Americans, whose diets generally do not include traditional African foods (Hoekstra *et al.*, 2008), suggests a strong influence of genetic factors. We found that our PRS based on 26 top SNPs had a weak predictive value in non-EU populations, despite the fact that the twinning rates were not adjusted for MZ twins or for maternal age. We expect this prediction may improve when it is feasible to account for these effects and for the different patterns of LD between lead SNPs and causal alleles in EU and other ancestry groups. Nevertheless separate and sufficiently powered GWAS studies of DZ twinning in African and Asian populations are highly desirable.

As recently as the 1950s the mean survival from a twin pregnancy in England and Wales was lower than that of singletons (Bulmer, 1970) and even today the cost of twin births, including their greater perinatal complications, is 4-fold that of singleton births (Chambers *et al.*, 2014). There has been extensive work on the genetics of twinning and fecundity in sheep and cattle (Galloway *et al.*, 2000; Wilson *et al.*, 2001; Monniaux, 2016), where litter size is an important economic trait, as well as in other mammals with higher litter sizes, notably marmosets (Harris *et al.*, 2014). The genes that we identified in humans (*FSHB*, *SMAD3*, *GNRH1*, *FSHR*) are part of the hypothalamic-pituitary-ovarian signaling axis, which is central to successful reproduction in mammalian species. However, to our knowledge, two of our top finds, *ZFPM1* and *IPO8*, have not been identified as involved in fecundity in other animals.

The gap between the common SNP-based heritability and twin-family heritability estimates implies that there are more variants to be identified, and indeed we observed many promising candidates not far below our threshold for genome-wide significance, indicating the need to expand sample size in future studies. The low heritability of twinning presumably reflects the many stochastic hazards faced by a twin pregnancy between ovulation and delivery of live twins. From ultrasound data, it is estimated that only 50% of twin conceptions result in liveborn twins (Samuels, 1988) so it is likely that the heritability of multiple ovulation, if we could assess it directly on the scale required (Martin *et al.*, 1991), would be higher than that of multiple births. The heritability of DZ twinning in humans is similar to estimates reported for the heritability of litter size in sheep (0.08–0.21) (Waldron and Thomas, 1992; Dewi *et al.*, 1996; Janssens *et al.*, 2004). Estimates of the heritability of ovulation rate in sheep, measured by counting corpora lutea at laparoscopy are considerably higher (0.34–0.58) (Hanrahan, 1990; Dewi *et al.*, 1996) than for twinning, suggesting GWAS for ovulation rate would have a greater power to detect genetic factors influencing DZ twinning, but this is difficult to measure at a large scale in humans. There may also be processes other than ovulation rate involved in DZ twinning, such as the capacity to carry a double pregnancy to term.

We identified only a few of the genes in the top pathway identified in the VEGAS2 pathway analysis (Supplementary Table S5), “Reactome hormone ligand binding receptors” (*P* = 3.6 × 10^−11^). This pathway includes the genes, *TSHB*, *FSHR*, *GNRHR*, *CGA*, *GNRH1*, *FSHB*, *TSHR*, *LHB*, and *GNRH2*, of which we detected only *FSHB, FSHR* and *GNRH1* in our GWAS, with the others producing little or no signal. While this may be due to technical issues (poor tagging, low power, limited genetic variation, or poor curation of pathways), it is also consistent with the between-species variations in the regulation of this pathway. Note also that this pathway does not include *IPO8* or *ZFPM1*. In this connection, we must remember that our results were obtained exclusively from samples of European ancestry. When sample sizes permit, it would be interesting to test whether the genetic architecture of DZT varies between the major ancestry groups that differ so greatly in DZ twinning rates.

## Conclusions

This new meta-analysis of genetic association studies of mothers of spontaneous DZ twins, as well as of DZ twins themselves, finds at least four new gene loci, *GNRH1/DOC5, FSHR/STON1-GTF2A1L/LHCGR*, *IOP8/CAPRIN2*, and *ZFPM/MIR5189*, in addition to two loci *SMAD3/*RP11-342M21.2 and *FSHB/ARL14EP* already known to influence DZ twinning in Europeans. These loci also appear to be generalizable in part to non-European populations and may explain some of the global variation in DZ twinning rates. All six genes have plausible roles in female reproduction and receive both *in vivo* and *in vitro* functional support. These results illuminate genetic influences on this variation in female fecundity and may in time suggest new strategies to tackle female infertility.

## Supplementary Material

dead247_Supplementary_Data_File_S1

dead247_Supplementary_Figure_S1

dead247_Supplementary_Figure_S2

dead247_Supplementary_Figure_S3

dead247_Supplementary_Figure_S4

dead247_Supplementary_Figure_S5

dead247_Supplementary_Figure_S6

dead247_Supplementary_Figure_S7

dead247_Supplementary_Figure_S8

dead247_Supplementary_Figure_S9

dead247_Supplementary_Figure_S10

dead247_Supplementary_Table_S1

dead247_Supplementary_Table_S2

dead247_Supplementary_Table_S3

dead247_Supplementary_Table_S4

dead247_Supplementary_Table_S5

dead247_Supplementary_Table_S6

dead247_Supplementary_Table_S7

dead247_Supplementary_Table_S8

dead247_Supplementary_Table_S9

dead247_Supplementary_Table_S10

dead247_Supplementary_Table_S11

dead247_Supplementary_Table_S12

dead247_Supplementary_Table_S13

dead247_Supplementary_Table_S14

dead247_Supplementary_Table_S15

dead247_Supplementary_Table_S16

## Data Availability

The data underlying this article are available in the NHGRI-EBI Catalog of human genome-wide association studies, at https://www.ebi.ac.uk/gwas/.
