# Supplementary material for: Genome-wide association study meta-analysis of dizygotic twinning illuminates genetic regulation of female fecundity
Source: Hum Reprod. 2023 Dec 5;39(1):240–57. doi: 10.1093/humrep/dead247 (PMC10767824; doi:10.1093/humrep/dead247)
Supplement: dead247_Supplementary_Data_File_S1 [file dead247_supplementary_data_file_s1.docx]

**Supplementary Data File S1. Supplementary materials and methods**

**Cohorts’ details on phenotyping, genotyping and quality control (QC)**

**Australia (including New Zealand, Belgium and Utah):** Two groups of special interest used in PRS analysis were genotyped, QC’d, and imputed alongside the main QIMR MoDZT group genotyped according to the process described in Gordon et al. (2023): (a) a Belgian sample of 40 MoDZTs from 13 multiplex families collected in the 1990s by Robert Vlietinck and Catherine Derom (Derom *et al.*, 2006); (b) a sample of 11 MoDZT from two multiplex families from the Utah Mormon Database, collected by Sue Healey (QIMR) in collaboration with Lisa Cannon-Albright (Univ Utah) in 1992 (Cannon-Albright *et al.*, 1994). Full details of recruitment, genotyping and imputation for the Australian plus New Zealand sample are given in Gordon et al. (2023).

**Netherlands Twin Register:** Genotyping was carried out across multiple platforms. Samples were removed if DNA sex did not match with expected sex, if the Plink heterozygosity F statistic was <-0.10, or > 0.10, or if the genotyping call rate was <.090. Palindromic AT/GC SNPs with an MAF range between 0.4 and 0.5 were removed. SNPs were removed if MAF <0.01, if the HWE p-value < 1×10^-5^, if call rate < 0.95, or if the N Mendel errors > 20.  These criteria were applied to each genotyping platform. After imputation, the datasets of each genotyping platform were merged and QC was repeated. For each platform, the data were position- and strand-aligned with the GONL reference set V4. SNPs that had a difference in allele frequency > 0.10, or had mismatching alleles with this reference panel were removed in this step (see Smit et al. (Smit *et al.*, 2020). All platform data were merged into a single dataset keeping all QCed SNPs of each platform (N=1.78M with  ~10.6k SNPs that all platforms have in common). Data were then cross-platform phased and imputed using MACH-ADMIX to predict the missing SNP genotypes in each platform as compared to other platforms, based on the complete GoNL reference panel haplotypes for the SNPs that were present in at least one platform (the ~1.78M). Participants with non-Dutch ancestry were defined based on PCA by projecting 10 PCs from 1000G reference Phase 3v5. We filtered on population based and sample MAF (0.03). Allele frequency differences between 1000G reference and sample over 0.20 were removed. Genotype imputation to the HRC 1.1 (~40m SNPs) and 1000G Phase 3 version 5 (~49m SNPs) reference panels was done on the cross-platform imputed data.

**Finnish Twin Cohort study:** Chip genotyping were done using Illumina Human610-Quad v1.0 B, Human670-QuadCustom v1.0 A, Illumina HumanCoreExome- (12 v1.0 A, 12 v1.1 A, 24 v1.0 A, 24 v1.1 A, 24 v1.2 A) and Affymetrix FinnGen Axiom arrays. The algorithm for genotype calling were Illumina’s GenCall for all HumanCoreExome chip genotypes, Illuminus for 610k & 670k chip genotypes and AxiomGT1 for Affymetrix chip genotypes. On Illumina arrays where genotypes were called to Illumina’s TOP strand, strands were flipped to forward strand using strand files generated by Will Rayner (https://www.well.ox.ac.uk/~wrayner/strand/). Genotype quality control were done in three batches (batch1: 610k+670k, batch2: HumanCoreExome and batch3: Affymetrix chip genotypes). Variants with call rate below 97.5% (batch1 and batch3) or 95% (batch2), samples with call rate below 98% (batch1) or 95% (batch2 and batch3), variants with minor allele frequency below 1% with Hardy-Weinberg Equilibrium p-value lower than 1e-06 were removed. Also samples from all batches with heterozygosity test method-of-moments F coefficient estimate value below -0.03 or higher than 0.05 (batch1 and batch2) or ±4SD from the mean (batch3) were removed along with the samples which failed sex check or were among the multi-dimensional scaling principal component analysis outliers. Total amount of genotyped variants after quality control were 486102 (batch1), 244545 (batch2) and 402933 (batch3) with the following number of samples remaining for imputation: 2617 (batch1), 5328 (batch2) and 8218 (batch3). Pre-phasing were performed using Eagle v2.4 (Loh *et al.*, 2016) and imputation with Minimac4 v1.0.0 (Fuchsberger *et al.*, 2015) using TOPMed Imputation Server (Das *et al.*, 2016). Genotypes of all batches were imputed to TOPMed release 2 reference panel (Taliun *et al.*, 2021). The final study sample were extracted from the data where all batches of imputed data were merged.

**Swedish Twin Registry:** The genotyping platform was Illumina OmniExpress imputed to 1000G Phase 1 version 3 using mach1 v1.0.18.c.

**Follow-up analyses**

**Combining GWAS results of true cases (i.e., MoDZTs) with proxy cases (being “part of a multiple birth”) by weighting the latter.**

In combining the GWA results for MoDZT with ‘being DZT’, the expectation is that the effect sizes (β) are halved in being twins. For a continuous phenotype (e.g. the liability to having twins), the regression analysis is straightforward in the MoDZT: regress liability on genotype (coded as 0,1,2). This will give the test of association and the estimate of additive genetic variance attributable to the genotype. We write this as:

Lm = β0 + β1*GVm + e

Lm = β0 + β1*(Am1 + Am2) + e

where Lm is the liability in mothers, GVm is the genetic variant in mother (coded 0,1,2) and Am1 and Am2 represent mother’s alleles coded as 0,1,2, where Am1+Am2 = GVm.

In the offspring, we ask "are you a DZ twin?" (0, 1), and first consider again the liability:

Lo = g0 + g1*GVo + e

Lo = g0 + g1*(Am + Af) + e

Lo = g0 + g1*(Am) + 0*Af + e

where Lo = liability in offspring; GVo is the genetic variant in the offspring; Am represents maternal allele and Af paternal allele and GVo = Am + Af (m=mother f=father; often we cannot assign parental origin). We assume that the paternal Af allele is not associated with the liability.

Supposing the liability is standardized var(Lm) = var (Lo) = 1, then

Lm = β0 + β1*(Am1 + Am2) + e = β0 + β1*Am1 + β1*Am2 + e

β1 = cov(Lm,Am1+Am2) = cov(Lm,Am1)+cov(Lm,Am2)

Lo = g0 + β1*Am + 0*Af + e

Lo = g0 + f1*(Am + Af) + e

cov(Lo, Am+Af) = cov(Lo, Am) + cov(Lo, Af) = cov(Lo, Am) + 0

var(Am+Af) = 1 (standardized)

f1 = cov(Lo, Am+Af) = cov(Lo, Am) = 1/2*b1

So, the raw regression coefficient obtained from analysis 2 (GWAS "are you a DZ twin") will produce f1, the raw regression coefficient obtained from analysis 1 (GWAS "are you the mother of DZ twins") will produce β1, where f1 = 1/2β.

In the meta-analysis of the two phenotypes, given β1 + se(β1) from analysis 1 and f1 and se(f1) from analysis 2, we transform f1 by f1*2 and get the standard error of f1*2 by means of the delta method, that is

sqrt(se(f1)^2^ * (d(2*f1)/dx)^2^ ),

where d(2*f1)/dx = 2 (1^st^ order derivative).

The switch to logistic regression does not change this. A numerical check follows below:

Numerical logistic regression for sample size 1,000,000 families

Phenotype: "are you mother of DZ" (0/1), mother genotype = predictor (coding 0,1,2) and father genotype = predictor (coding 0,1,2)

Estimate Std. Error z value Pr(>|z|)

Mother (Intercept) -2.187024 0.008572 -255.15 <2e-16 ***

G[1:n2, 1] 0.100342 0.005218 19.23 <2e-16 *** parameter β1

Father (Intercept) -2.040101 0.008289 -246.130 <2e-16 ***

father G[1:n2, 2] 0.003418 0.005111 0.669 0.504

Phenotype : "are you a DZ twin", genotype offspring predictor (coding 0,1,2)

Estimate Std. Error z value Pr(>|z|)

Offspring (Intercept) -2.112701 0.008430 -250.622 <2e-16 ***

G[1:n2, 3] 0.051529 0.005164 9.978 <2e-16 *** parameter f1

Thus,

β1= .1003, se(β1) = .0052

transform f1: f1 = .0515* 2 = .103

se(f1) = sqrt(4*.005164^2) = .0103

**Polygenic risk score (PRS) analyses in NTR and Australia:** We employed LDPRED (Lloyd-Jones *et al.*, 2019) to obtain PRS based on the summary statistics from the full GWAMA minus the NTR sample itself. Prior to PRS calculations on the HRC imputed data, we excluded insertion/deletion variants, those with strand ambiguity or low-imputation quality (R2<0.3), MAF < 0.01, HWE p-value < 0.0001. Using unrelated individuals from the NTR cohort the genotypes and summary statistics were coordinated. Then LDPRED was run with a 250 kb LD window for the top 1.0, 0.5, 0.3, 0.2, 0.1, 0.05, 0.03, 0.01, 0.005, 0.003, 0.001, 0.0005, 0.0003, 0.0001 proportions of SNPs in the summary statistics. For each of the resulting weighted GWAS effect sizes, the DZT PRS for the NTR sample of cases and controls were calculated using PLINK 1.9 (Chang *et al.*, 2015) (Supplementary Fig. S2). With Pearson correlations the optimal predicting proportion was determined to be 0.001. Subsequently, using this PRS the odds ratio of giving birth to DZ twins was plotted by PRS decile in Supplementary Fig. S3.

We also calculated PRS for two special out-of-sample studies of DZT dense pedigrees in Belgium (21 cases) and Utah (10 cases) and compared them with the distribution of DZT PRS in Australian cases and controls. While these did not show elevated PRS at the 𝛼 < 0.001 level relative to controls (Supplementary Fig. S4 and Supplementary Table SⅥ), Belgian cases showed increased frequencies of the risk alleles defined as the top SNPs in the best 12 genes from the gene-based test, significantly so for *GNRH1* (p=.03, Supplementary Table SⅥ). However, there was no discernible pattern of increase in the two Utah pedigrees (not shown).

**Polygenic scores for the PheWAS:** Polygenic risk scores (PRS) for DZ twinning were calculated for each individual using the PRS-CS software package, which applies a Bayesian continuous shrinkage model to SNP beta estimates to adjust for linkage disequilibrium(Ge *et al.*, 2019). With this framework, no p-value thresholds are required to generate PRS. Beta estimates were drawn from the summary statistics of the full MoDZT+DZT GWAMA (Table Ⅱ). To maximize our ability to assess the full polygenicity of DZT on predicting DZT case status, SNPs (n = 782,190 matching the hapmap panel) from the summary statistics dataset were used to generate DZT PRS for a total of 37,032 genotyped females of European descent in BioVU. Genotyping and QC of this sample have been described elsewhere (Denny *et al.*, 2010; Ruderfer *et al.*, 2020).

**PheWAS:** In the genotyped BioVU sample, a logistic regression model was fitted to each of 1,095 case/control phenotypes to estimate the odds of each diagnosis given the DZT polygenic score in females, after adjustment of median age across the EHR and the top 10 principal components of ancestry. The 1,095 disease phenotypes included 35 infectious diseases, 91 neoplasms, 101 endocrine/metabolic diseases, 34 hematopoietic diseases, 55 mental disorders, 54 neurological disorders, 66 sense organs, 121 circulatory system disorders, 64 respiratory diseases, 107 digestive diseases, 102 genitourinary diseases, 24 pregnancy complications, 62 dermatologic disorders, 75 musculoskeletal disorders, 20 congenital anomalies, 28 symptoms, and 56 injuries/poisonings. We required the presence of at least two International Classification of Disease (ICD) codes that mapped to a PheWAS disease category (Phecode Map 1.2 (https:// phewascatalog.org/phecodes) to assign ‘case’ status. PheWAS analyses were run using the PheWAS R package (Carroll *et al.*, 2014).

**Genetic correlation analyses:** We performed pairwise genetic correlation analysis between DZ twinning and previously published phenotypes, using bivariate LD-score (LDSC) regression (Bulik-Sullivan *et al.*, 2015a, 2015b). LDSC estimates the genetic correlation (between -1 and 1) between two traits from GWAS summary statistics. To reduce bias due to multiple genotyping platforms, correlations were calculated based on a common set of HapMap3 variants. We performed our analyses on the cloud platforms Complex Trait Genomics Virtual Lab (CTG-VL; [vl.genoma.io](http://vl.genoma.io/)) (Cuellar-Partida *et al.*, n.d.) and LD-hub ([www.ldhub.org](http://www.ldhub.org/)) (Zheng *et al.*, 2017), both of which aggregate summary statistics for GWAS on hundreds of traits. Additionally, correlations with testosterone, estradiol and SHBG were calculated locally using LDSC. We have tested over 2180 traits based on publicly available data. We focused here on health-related and anthropometric traits, the two categories most likely related to fertility and reproduction.

Full results for all nominally significant correlations are shown in Supplementary Table SⅦ for CTG-VL results and Supplementary Table SⅧ for LD-hub results. CTG-VL yielded nominally-significant genetic correlations with 189 traits (p<0.05), and LD-hub yielded only 30 nominally significant genetic correlations. CTG-VL has more traits in its database, while LD-hub is generally more curated but less updated. No multiple-testing correction has been applied. Rgs with *in vivo* hormone levels are in Supplementary Table SⅨ and with cancer and smoking in Supplementary Table SⅩ.

**Heritability:** Methods for the estimation of biometric (family/twin based) heritability are described in the main text. Our SBayesS analysis (see Evidence of Selection, below) also provided an estimate of the SNP-based heritability of DZT ~0.5% (S.E. 0.05%) on the observed binary scale (consistent with the estimate from LD score regression: 0.4% (S.E. 0.009%)), that corresponds to an estimate of ~2.4% (S.E. 0.3%) on the continuous liability scale, assuming a population prevalence of ~1% for DZT.

**Look ups: In vivo hormone assays**

**NESDA**: Data were derived from the Netherlands Study of Depression and Anxiety (NESDA), serum levels on 2133 unrelated subjects were measured as detailed in (de Wit *et al.*, 2021) by proteomic profiling. A detailed description of the sample characteristic can be found in Bot et al. (Bot *et al.*, 2015)

**deCODE:** Hormone testing was undertaken primarily to investigate possible gonad impairment. Serum follicle-stimulating hormone (FSH), luteinizing hormone (LH), estradiol, testosterone and sex hormone-binding globulin (SHBG) levels were measured by electrochemiluminescence immunoassays, using reagents, calibrators and analytical instruments from Roche Diagnostics GmbH according to the manufacturer’s instructions.

**TwinsUK:** Plasma levels of DHEAS, FSH, LH, oestradiol, progesterone, prolactin, SHBG and testosterone were measured on 2913 adult twins (294 males) by commercial ElectroChemiLuminescent immunoassays on a Modular Analytics E170 analyser (Roche Diagnostics GmbH, Mannheim, Germany) using the prescribed assay calibrators and performed according to the manufacturer’s protocol. Details of the immunoassays can be found in Ruth et al. (Ruth *et al.*, 2016). Look-ups of our top SNPs were performed for FSH, LH, SHBG, testosterone and estradiol.

**UK Biobank:** Testosterone, Estradiol and Sex Hormone Binding Globulin were assayed in 425,097 UKBiobank study participants, as detailed in Ruth et al. (Supplementary Table SⅫ) (Ruth *et al.*, 2020) The effect sizes and significance of top SNPs for DZT were extracted from the GWAS summary statistics for these hormones which are available online.

**In silico functional annotations: eQTL, GTEX, SMR**

To assess if any of the SNPs in the five independent loci were also associated with expression of genes in the region, we tested for overlap between SNPs in LD with lead SNPs (r2>0.7) and SNPs significantly associated with gene expression in eQTL datasets including endometrial eQTLs generated using both microarray (Fung *et al.*, 2017) and RNA-sequencing (RNA-Seq) data (Mortlock *et al.*, 2020) (FDR<0.05), 49 tissues from GTEx (Gamazon *et al.*, 2018) (p<1x10-5) and a large blood eQTL database, eQTLGen (Võsa *et al.*, 2021) (p<3.93x10-10). We also tested for overlap with endometrial mQTLs (Mortlock *et al.*, 2019) (FDR<0.05) and a large blood mQTL dataset21 (p<5x10-8). SNPs within genes identified as significantly associated with DZ twinning in the gene-based analysis were also investigated for overlap with QTLs.

**TWAS**

We applied the python-based tool MetaXcan to perform a Transcriptome-Wide Association Scan (TWAS) for DZ twinning. MetaXcan employs cis-eQTL information to impute the genetically regulated gene-expression based on GWAMA results (Gamazon *et al.*, 2015; Barbeira *et al.*, 2018). The analysis was performed as described in the tutorial (https://github.com/hakyimlab/MetaXcan). TWAS utilizes genotype and eQTL reference data to train prediction models that can be used to impute gene expression in independent datasets for which only genotype data are available. In the Joint Tissue Imputation (JTI) extension, covariance information from transcriptomic information across tissues improves the imputation of gene-expression based on multi-tissue models (Zhou *et al.*, 2020). The JTI models were derived from the GTEx database (Consortium, 2020) and were downloaded from Gamazon and Zhou (Gamazon *et al.*, 2018; Zhou *et al.*, 2020).

Since gene-expression levels vary across tissues, eight tissues involved with processes concerning fertility or hormonal regulation were included in the TWAS analysis, i.e., breast, hypothalamus, ovary, pituitary gland, testis, uterus, vagina and whole blood. The eight tissues had relatively large sample sizes in the GTEx database, ranging from 129 for uterus to 670 for whole blood samples. We calculated two different significant levels based on Bonferroni corrections: a tissue-specific value and a tissue-wide value. The tissue-specific value was based on the number of genes that were imputed in a specific tissue model, while the tissue-wide value was based on the total number of genes for all models combined (Supplementary Tables SXIII and XIV).

**Evidence of selection against DZT**

We applied two approaches to test if the DZT has been undergoing natural selection. Our first approach is based on the SBayesS method , which quantifies the relationship between the variance of allele substitution effects (β) and frequency (p) across all SNPs through the following model: var(β)=〖[2p(1-p)]〗^S, where S is the parameter of interest. A large negative value of S means that low frequency variants contribute more to the heritability of DZT than more common SNPs, which is expected under negative selection. We performed a SBayesS analysis using 1.1 M common (MAF>1%) HapMap 3 SNPs and found a significant S~-0.69 (S.E. 0.17). Interestingly, this estimate is similar to that observed across fertility-related traits in the UK Biobank(Zeng *et al.*, 2021). Our SBayesS analysis also provided an estimate of the SNP-based heritability of DZT ~0.5% (S.E. 0.05%) on the observed binary scale (consistent with the estimate from LD score regression: 0.4% (S.E. 0.009%)), that corresponds to an estimate of ~2.4% (S.E. 0.3%) on the continuous liability scale, assuming a population prevalence of ~1% for DZT.

Our second approach uses singleton density scores (SDS) as a measure of recent selection (Field *et al.*, 2016). More specifically, we tested if the density of singletons (i.e., variants observed in only 1 study participant), which indicates recent changes in allele frequency, is different at DZT associated loci as compared to the rest of the genome. After Bonferroni correction to account for the number of loci tested, we could not identify any DZT-associated SNP with a significant SDS signal. The strongest signal was observed on chromosome 11 at rs7951733, where the SDS of the DZT-increasing allele (iSDS) is ~2.5 SD lower (equivalent to P=0.01) than the rest of the genome. Next, we tested if, on average across loci, iSDS were different from zero, which would provide evidence of directional selection. We found a marginally significant negative correlation between iSDS and strength of association between SNPs and DZT (r=-0.38, jackknife S.E.=0.17, P=0.026; Supplementary Fig. S10). This result suggests that the frequencies of DZT-increasing alleles have decreased over the past ~100 generations in ancestors of the contemporary UK population.

To test further if this selection signal is truly polygenic, we used the LD score test proposed by Field et al.(Field *et al.*, 2016). In brief, this test quantifies the relationship between LD scores on the one hand and the covariance between DZT Z-scores and SDS on the other hand. The underlying assumption of this test is that the magnitude of the covariance between DZT Z-scores and SDS should increase with LD scores if selection is polygenic, while it would be independent of LD scores if the signal is driven by stratification alone. We observed a negative association between LD scores and the covariance between DZT Z-scores and SDS. However, standard errors are too large to make a clear conclusion regarding the polygenicity of this signal (r=-0.03, jackknife S.E. 0.02).

**Zebrafish methods**

Zebrafish fertility quantification: Adult fish (between 4 and 6 months old) of the appropriate genotype were crossed as indicated. For each cross, clutch size and embryos survival at 24 hours post fertilization were calculated. Each cross was repeated using different animals a minimum of 6 times, obtaining clutches containing between 111 and 378 eggs each.

Whole mount in-situ hybridization: To generate anti-sense probes, DNA fragments were obtained by PCR using Phusion™ High-Fidelity DNA Polymerase (Thermo Scientific™) and the following primers (5’->3’): forward: TTACAGTGTCGTGGACGTGG, reverse: CCTGCTCCGTGTTCAGAGTT (amplicon seize 691bp). Total cDNA of 2 dpf Zebrafish was used as a template. PCR fragment was cloned into the pCRII-TOPO vector (Invitrogen) according to manufacturer’s instructions. The generated plasmid used was sequenced for confirmation. Anti-sens DIG-RNA-labeled probes was in-vitro transcribed using the RNA Labeling Kit (Roche Diagnostics Corporation) according to manufacturer's instructions.

Whole-mount digoxigenin *in-situ* hybridization was performed according to standard protocols (Thisse and Thisse, 2008). Briefly, after fixation and permeabilization, the sample were immersed in a solution of DIG-labeled probe overnight at 68°C. Excess probes were removed by several washes. An alkaline phosphatase-conjugated anti-DIG-AP Fab fragments antibody (Roche) was used against digoxigenin and a chromogenic NBT-BCIP substrate (Roche) was used to detect the hybridization.

Samples were imaged on a stereoscope Leica MZ10F, with Leica DFC310FX camera

Quantitative RT-PCR: Total RNA was prepared from 64 cells stage embryos (100-150 embryos per sample) with TRIzol reagent (ThermoFisher Scientific) and TURBO DNA-free treatment (ThermoFisher Scientific). 1 μg of total RNA was retrotranscribed using random hexamers and the SuperScript III First-Strand Synthesis system (ThermoFisher Scientific) according to the manufacturer’s instructions. For q-RT-PCR, SYBR Green PCR Master Mix (ThermoFisher Scientific) was used according to manufacturer’s instructions and the PCR reaction was performed on an STEPONE Plus Real-Time PCR System instrument (ThermoFisher). *Ef1a* and *RPL13a* were used as reference genes as reported previously (Tang *et al.*, 2007). All assays were performed in biological triplicate using 11.25 ng of cDNA per reaction. The mean values of triplicate experiments were calculated according to the delta*CT* quantification method. The following primers were used for Ipo8 quantification (5’->3’) : forward : TCCGTCGCTCCCACAGGAAA, reverse : TCGCAGTCCAGTGGCGTACT (amplicon seize 122 bp).

**Supplementary Web Resources**

MetaXcan, <https://github.com/hakyimlab/MetaXcan>

JTI, <https://zenodo.org/record/3842289#.YX5err1BwlI>

GCTB:<https://cnsgenomics.com/software/gctb/#Overview>

LD matrices:<https://zenodo.org/record/3350914#.XyFfnC17GBo>

LD scores:<https://data.broadinstitute.org/alkesgroup/LDSCORE>

SDS:<https://web.stanford.edu/group/pritchardlab/UK10K-SDS-values.zip>

**Supplementary References**

Barbeira AN, Dickinson SP, Bonazzola R, Zheng J, Wheeler HE, Torres JM, Torstenson ES, Shah KP, Garcia T, Edwards TL, *et al.* Exploring the phenotypic consequences of tissue specific gene expression variation inferred from GWAS summary statistics. *Nat Commun* [Internet] 2018;**9**:1825.

Bot M, Chan MK, Jansen R, Lamers F, Vogelzangs N, Steiner J, Leweke FM, Rothermundt M, Cooper J, Bahn S, *et al.* Serum proteomic profiling of major depressive disorder. *Transl Psychiatry* [Internet] 2015;**5**:e599.

Bulik-Sullivan B, Finucane HK, Anttila V, Gusev A, Day FR, Loh P-R, Consortium R, Consortium PG, 3 GC for AN of the WTCCC, Duncan L, *et al.* An atlas of genetic correlations across human diseases and traits. *Nat Genet* [Internet] 2015a;**47**:1236–1241.

Bulik-Sullivan BK, Loh P-R, Finucane HK, Ripke S, Yang J, Consortium SWG of the PG, Patterson N, Daly MJ, Price AL, Neale BM. LD Score regression distinguishes confounding from polygenicity in genome-wide association studies. *Nat Genet* [Internet] 2015b;**47**:291–295. 1] Program in Medical and Population Genetics, Broad Institute of MIT and Harvard, Cambridge, Massachusetts, USA. [2] Analytical and Translational Genetics Unit, Department of Medicine, Massachusetts General Hospital and Harvard Medical School, Boston, Ma.

Cannon-Albright LA, Thomas A, Goldgar DE, Gholami K, Rowe K, Jacobsen M, McWhorter WP, Skolnick MH. Familiality of cancer in Utah. *Cancer Res* [Internet] 1994;**54**:2378–2385.

Carroll RJ, Bastarache L, Denny JC. R PheWAS: data analysis and plotting tools for phenome-wide association studies in the R environment. *Bioinformatics* [Internet] 2014;**30**:2375–2376.

Chang CC, Chow CC, Tellier LC, Vattikuti S, Purcell SM, Lee JJ. Second-generation PLINK: rising to the challenge of larger and richer datasets. *Gigascience* [Internet] 2015;**4**:7. Oxford Academic.

Consortium Gte. The GTEx Consortium atlas of genetic regulatory effects across human tissues. *Science (80- )* [Internet] 2020;**369**:1318–1330.

Cuellar-Partida G, Lundberg M, Kho PF, D’Urso S, Gutierrez-Mondragon LF, Hwang L-D. Complex-Traits Genetics Virtual Lab: A community-driven web platform for post-GWAS analysesAvailable from: http://dx.doi.org/10.1101/518027.

Das S, Forer L, Schönherr S, Sidore C, Locke AE, Kwong A, Vrieze SI, Chew EY, Levy S, McGue M, *et al.* Next-generation genotype imputation service and methods. *Nat Genet* 2016;**48**:1284–1287.

Denny JC, Ritchie MD, Basford MA, Pulley JM, Bastarache L, Brown-Gentry K, Wang D, Masys DR, Roden DM, Crawford DC. PheWAS: demonstrating the feasibility of a phenome-wide scan to discover gene-disease associations. *Bioinformatics* [Internet] 2010;**26**:1205–1210.

Derom C, Jawaheer D, Chen W V, McBride KL, Xiao X, Amos C, Gregersen PK, Vlietinck R. Genome-wide linkage scan for spontaneous DZ twinning. *Eur J Hum Genet* [Internet] 2006;**14**:117–122.

Field Y, Boyle EA, Telis N, Gao Z, Gaulton KJ, Golan D, Yengo L, Rocheleau G, Froguel P, McCarthy MI, *et al.* Detection of human adaptation during the past 2000 years. *Science* [Internet] 2016;**354**:760–764. Science.

Fuchsberger C, Abecasis GR, Hinds DA. minimac2: faster genotype imputation. *Bioinformatics* [Internet] 2015;**31**:782–784.

Fung JN, Girling JE, Lukowski SW, Sapkota Y, Wallace L, Holdsworth-Carson SJ, Henders AK, Healey M, Rogers PAW, Powell JE, *et al.* The genetic regulation of transcription in human endometrial tissue. *Hum Reprod* [Internet] 2017;**32**:893–904.

Gamazon ER, Segrè A V, Bunt M van de, Wen X, Xi HS, Hormozdiari F, Ongen H, Konkashbaev A, Derks EM, Aguet F, *et al.* Using an atlas of gene regulation across 44 human tissues to inform complex disease- and trait-associated variation. *Nat Genet* [Internet] 2018;**50**:956–967.

Gamazon ER, Wheeler HE, Shah KP, Mozaffari S V, Aquino-Michaels K, Carroll RJ, Eyler AE, Denny JC, Consortium Gte, Nicolae DL, *et al.* A gene-based association method for mapping traits using reference transcriptome data. *Nat Genet* [Internet] 2015;**47**:1091–1098.

Haak W, Forster P, Bramanti B, Matsumura S, Brandt G, Tänzer M, Villems R, Renfrew C, Gronenborn D, Alt KW, *et al.* Ancient DNA from the first European farmers in 7500-year-old Neolithic sites. *Science (80- )* [Internet] 2005;**310**:1016–1018.

Lloyd-Jones LR, Zeng J, Sidorenko J, Yengo L, Moser G, Kemper KE, Wang H, Zheng Z, Magi R, Esko T, *et al.* Improved polygenic prediction by Bayesian multiple regression on summary statistics. *Nat Commun* [Internet] 2019;**10**:5086.

Loh P-R, Danecek P, Palamara PF, Fuchsberger C, A Reshef Y, K Finucane H, Schoenherr S, Forer L, McCarthy S, Abecasis GR, *et al.* Reference-based phasing using the Haplotype Reference Consortium panel. *Nat Genet* [Internet] 2016;**48**:1443–1448.

Mortlock S, Kendarsari RI, Fung JN, Gibson G, Yang F, Restuadi R, Girling JE, Holdsworth-Carson SJ, Teh WT, Lukowski SW, *et al.* Tissue specific regulation of transcription in endometrium and association with disease. *Hum Reprod* [Internet] 2020;**35**:377–393.

Mortlock S, Restuadi R, Levien R, Girling JE, Holdsworth-Carson SJ, Healey M, Zhu Z, Qi T, Wu Y, Lukowski SW, *et al.* Genetic regulation of methylation in human endometrium and blood and gene targets for reproductive diseases. *Clin Epigenetics* [Internet] 2019;**11**:49.

Ruderfer DM, Walsh CG, Aguirre MW, Tanigawa Y, Ribeiro JD, Franklin JC, Rivas MA. Significant shared heritability underlies suicide attempt and clinically predicted probability of attempting suicide. *Mol Psychiatry* [Internet] 2020;**25**:2422–2430.

Ruth KS, Campbell PJ, Chew S, Lim EM, Hadlow N, Stuckey BGA, Brown SJ, Feenstra B, Joseph J, Surdulescu GL, *et al.* Genome-wide association study with 1000 genomes imputation identifies signals for nine sex hormone-related phenotypes. *Eur J Hum Genet* [Internet] 2016;**24**:284–290.

Ruth KS, Day FR, Tyrrell J, Thompson DJ, Wood AR, Mahajan A, Beaumont RN, Wittemans L, Martin S, Busch AS, *et al.* Using human genetics to understand the disease impacts of testosterone in men and women. *Nat Med* [Internet] 2020;**26**:252–258.

Taliun D, Harris DN, Kessler MD, Carlson J, Szpiech ZA, Torres R, Taliun SAG, Corvelo A, Gogarten SM, Kang HM, *et al.* Sequencing of 53,831 diverse genomes from the NHLBI TOPMed Program. *Nature* [Internet] 2021;**590**:290–299.

Tang R, Dodd A, Lai D, McNabb WC, Love DR. Validation of zebrafish (Danio rerio) reference genes for quantitative real-time RT-PCR normalization. *Acta Biochim Biophys Sin (Shanghai)* [Internet] 2007;**39**:384–390. Acta Biochim Biophys Sin (Shanghai).

Thisse C, Thisse B. High-resolution in situ hybridization to whole-mount zebrafish embryos. *Nat Protoc* [Internet] 2008;**3**:59–69. Nat Protoc.

Võsa U, Claringbould A, Westra H-J, Bonder MJ, Deelen P, Zeng B, Kirsten H, Saha A, Kreuzhuber R, Yazar S, *et al.* Large-scale cis- and trans-eQTL analyses identify thousands of genetic loci and polygenic scores that regulate blood gene expression. *Nat Genet* [Internet] 2021;**53**:1300–1310.

Wit AE de, Giltay EJ, Boer MK de, Bosker FJ, Cohn AY, Nolen WA, Kaiser UB, Joffe H, Penninx BWJH, Schoevers RA. Plasma androgens and the presence and course of depression in a large cohort of women. *Transl Psychiatry* [Internet] 2021;**11**:. Transl Psychiatry.

Zheng J, Erzurumluoglu AM, Elsworth BL, Kemp JP, Howe L, Haycock PC, Hemani G, Tansey K, Laurin C, Consortium EG and LE (EAGLE) E, *et al.* LD Hub: a centralized database and web interface to perform LD score regression that maximizes the potential of summary level GWAS data for SNP heritability and genetic correlation analysis. *Bioinformatics* [Internet] 2017;**33**:272–279. MRC Integrative Epidemiology Unit, University of Bristol, Oakfield House, Bristol, UK. Genetic Epidemiology Group, Department of Health Sciences, University of Leicester, Leicester, UK. University of Queensland Diamantina Institute, Translational Research.

Zhou D, Jiang Y, Zhong X, Cox NJ, Liu C, Gamazon ER. A unified framework for joint-tissue transcriptome-wide association and Mendelian randomization analysis. *Nat Genet* [Internet] 2020;**52**:1239–1246. Division of Genetic Medicine, Department of Medicine, Vanderbilt University Medical Center, Nashville, TN, USA. zdangm@gmail.com. Vanderbilt Genetics Institute, Vanderbilt University Medical Center, Nashville, TN, USA. zdangm@gmail.com. Vanderbilt Genetic.


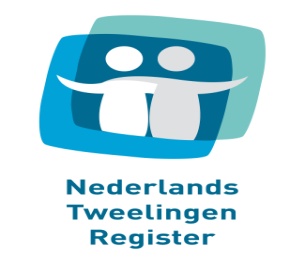

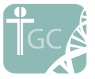
 **Twinning Genetics Consortium**

[www.twinningconsortium.org](http://www.twinningconsortium.org)

**Analysis Plan**

**Version:** 20 September 2017

**Filename:** TwinningGeneticsConsortium_AnalysisPlan_date.doc

**Attached file:** Study Descriptive_date.xlsx

- **Collaborating Investigators**
- *Data uploaded*

**Netherlands Twin Register**: Hamdi Mbarek, Jouke Jan Hottenga, Gonneke Willemsen, Conor Dolan, Dorret Boomsma

**Queensland Twin Registry**: Allan McRae, Scott Gordon, Dale Nyholt, Grant Montgomery, Nick Martin

**Finnish Twin Cohort**: Teemu Palviainen**,** Miina Ollikainen, Kauko Heikkila, Jaakko Kaprio

**Minnesota Center for Twin and Family Research:** Scott Vrieze, Mike Miller, Matt Mc Gue

**Twins Early Development Study**: Eva Krahpol, Robert Plomin

**Twins UK:** Hysi Pirro, Massimo Mangino, Tim Spector

**Swedish Twin Registry**: Nancy Pedersen, Patrik Magnusson

**Women’s Genome Health Study**: Samantha Schilit, Scott Gallagher, Daniel Chasman, Cynthia Morton

**Danish Twin Register:** Marianne Nygaard, Lene Christiansen, Kaare Christensen

**deCODE:** Stacy Steinberg, Hreinn Stefansson, Kari Stefansson

**Spanish Twin Register:** Juan R. Ordonana

1. Background

The purpose of the [Twinning Genetics Consortium](http://www.twinningconsortium.org/) (TGC) is to conduct meta-analyses of genome-wide association study data to identify genetic polymorphisms associated with the “Twinning” trait. Knowledge of these genes may identify key mechanisms controlling ovarian function and can provide a greater understanding of female fertility and infertility, the most common reproductive disorder.

This project is divided into two parts: Dizygotic twinning and Monozygotic twinning.

**Dizygotic twinning**

Early association studies of DZ twinning were with obvious candidate genes, including those for peptide hormones known to be involved in reproduction (*FSHB*, *FSHR*, *LHB*, inhibins, *GNRH* and *FST*) as well as less obvious ones including the fragile-X syndrome gene *FRAXA*, *PPARG* and *MTHFR*. As with candidate gene studies in most other complex traits, results have been mixed and only few replicable associations have been found. Many of these studies were also statistically underpowered given current ideas of gene effect sizes. It is now clearly necessary to take a more global approach to this problem, using genome wide association with a very large population to obtain adequate statistical power. We have collected over 35,000 individuals of European ancestry, representing the largest twins cohorts in the world. With this resource, we have performed the first genome-wide association study (GWAS) for being a mother of DZ twins in 1,980 mothers of spontaneous DZ twins and 12,953 controls of European ancestry from The Netherlands, US, and Australia and published our first findings in 2016 (DOI: 10.1016/j.ajhg.2016.03.008).

**Monozygotic twinning**

There is no clear evidence yet for the implication of hereditary factors in a genetic contribution to having monozygotic twins. However, based on work by Bruno Reversade (IMB Singapore) in some rare cases MZ twinning seems to be segregating in families. And although this is likely a rare allele with substantial effect Mendelian trait, given the enrichment that twin studies have in their data for MZ twins as well as DZ twins this might be the right sample to look for these specific mutations.

We aim here to conduct the first meta-analyses for gwas of DZ and MZ twinning.

1. Trait definition

**A. DZ twinning**

- - 1. **Being a “dizygotic twin”**

Cases : DZ twin

Controls: non DZ twin (mothers and fathers of MZ twins, mothers and fathers of singletons, case-mix or healthy controls)

This study design is suitable when only genotyped DZ twins without mothers genotypes are available from a single cohort

- - 1. **Being a “mother of dizygotic twin”**

Cases : unrelated mothers of DZ twins with documented history of spontaneous DZ twinning (*i.e.* no use of artificial reproduction techniques-ART)

Controls: mothers and fathers of MZ twins, mothers and fathers of singletons, case-mix or healthy controls

This study design is intended when both genotyped DZ mothers and DZ twins without mothers genotypes are available from a single cohort. The missing information for the ungenotyped mother can be inferred from the offspring. This study is most powerful and has the advantage of using all her offspring’s genotypes to infer her genotype rather than just the genotypes of the DZ twin.

**B. Monozygotic twinning**

The trait here is being a “monozygotic twin”

Cases**:** MZ twin

Controls: non MZ twin ( DZ twin, case-mix or healthy controls)

**C. Choice of controls**

- Large epidemiologic studies have found no etiological connection between DZ and MZ twinning. Thus, parents of MZ twins form useful controls and we shall use both mothers and fathers where available.

- We can use case-mix samples as controls, provided there is no etiologic link between the disease and DZ/MZ twinning. An advantage is that these collections are drawn from exactly the same populations, as the cohort used for the gwas so should minimize stratification effects.

- When using mothers of singletons as controls we should be aware that we need to check carefully the allele frequency comparing to MOTHDZT at least for the tops SNPs as we may detect loci that control the ability to carry a multiple pregnancy.

- A paper published recently by Kosova *et al.* 2012 showed some evidence for a paternal effect on fertility. Thus we excluded fathers of DZ from the control group for MOTHDZT gwas. We can run later the same gwas to test for this hypothesis.

1. Consortium opt-in procedure

To participate in the initiative, please complete the attached descriptive statistics summary file (STUDYdescriptivesDATE.xls) for your sample and send it to Hamdi Mbarek (h.mbarek@vu.nl) **by 01 October 2017**.

Please report statistics based on genotyped individuals who will be included in the GWAS and meta-analysis. If you have any questions about the phenotype definition, please contact Hamdi Mbarek ASAP. We will circulate a collaboration agreement in due time.

1. Sample exclusion criteria

- Mothers of DZ twins who received fertility treatment (**if it is a large group of sample, please run separate analyses with and without this group**). If information on fertility treatment is not available, please include only mothers who gave birth before 1967 (clomiphene introduction). This can be a little different in each country.
- Missing data on covariates
- Non-caucasian

1. Covariates

- Sex (except for MODZT gwas)
- PC’s, major principal components (or equivalent, if available) in each study

1. Genotypes & Imputation

We assume that all cohort followed the 1000 Genomes imputations approach developed for the GIANT consortium or used one the imputation server service below.

The panel for imputation is the **HRC 1.1**. If not available please use **1000G Phase III Version 5**. If you have used a version previous to this release, please redo your imputation with this dataset or in case this cannot be done in relevant time contact us for further information.

**Links:**

Michigan imputation server

<https://imputationserver.sph.umich.edu/index.html>

Sanger imputation server

<https://imputation.sanger.ac.uk/>

Please provide unfiltered results for autosomal SNPs, as filters on imputed markers and genomic control will be applied at the meta-analysis stage, unless faulty SNPs cannot be taken out based on R2, Info, MAF or HWE.

1. Models used to test association

The imputed genotypes should be used in a way that explicitly takes account of uncertainty in the imputed genotypes, e.g. the .mldose file from MACH or the genotype probability file output by IMPUTE. Directly measured genotypes should be used in place of imputed genotypes were possible.

Logistic regression model will be performed on all samples adjusted for appropriate covariates.

**Softwares**:

- PLINK 1.07 (for unrelated or also family based with the -- family command)

**A. GWAS DZ twinning**

1. **DZT *vs* controls**

Logit (DZT vs controls)= SNP + sex + 10 PC’s

1. **MOTHDZT *vs* controls**

Logit (MOTHDZT vs controls)= SNP + 10 PC’s

**B. GWAS MZ twinning**

1. **MZT *vs* controls**

Logit (MZT vs controls)= SNP + sex + 10 PC’s

1. Timeline for delivery of results

Please deliver the descriptive statistics for this phenotype by: **01 December 2017** (reported using the attached spreadsheet, ‘STUDYdescriptivesDATE.xls’). Please send by email to hmbarek@vu.nl or upload via sharespace.

Please deliver your GWAS results by: **15 December 2017**

1. Results file formats

**A. File formatting**

-Report results in tab-delimited txt-format and please use EXACTELY the column names AND ORDER as in the template below. Ensure all column names are written as they appear below- in lower case with underscores preserved.

-All numeric data can be specified in either scientific or decimal notation and should be 4 or 6 decimal places. P-values should be specified to 4 significant digits.

-Integer data should be supplied as a single integer number with no decimal point.

-Code missing values in any column as -999.

-No quotes should be used around any data cells or headers

-No row indices column or any other extra column should be provided.

-Remove monomorphic SNPs in your data and SNPs, for which no association data is available. Please supply a separate list for these snps in txt-format (see below for the format)

**Please always compress files before uploading! (preferably use gzip)**

- **markersnp**: Please use the markername exactly as it is represented in the imputation output (Build37 v3 1000 genomes). We will convert these to a common Id during the cleaning and meta-analysis.
- **Chr**: chromosome number
- **position:** basepair position according to Build37 v3 1000 genomes
- **strand**: strand (+/-) according to Human Reference Genome Built 37 (GRCh37)
- **n**: total number of individuals used in the analyses with genotypes and phenotypes for the specific SNP.
- **effect_allele**: coded/effect/predictor allele (a single character: “A” “C” “G” “T”)
- **other_allele**: control/reference/non-coded/ baseline allele (a single character: “A” “C” “G” “T”)
- **hwe_pval:** hardy-weinburg equilibrium test p-value
- **eaf**: allele frequency for **effect_allele** (numeric data, up to 3 decimal places)
- **beta**: effect size for each copy of the effect/coded allele (numeric data, with at least 4 decimal places)
- **se**: standard errors of beta (numeric data, with at least 6 decimal places)
- **p**: P value for the association between the marker and the outcome (4 significant figures)
- **imputation**: values (R2, proper_info or INFO) corresponding to the information content output from the association testing for imputed SNPs (numeric data, up to 2 decimal places. Do this for all SNPs including the ones genotyped.

**B. File naming scheme**

Please provide the output for your analysis as follow:

**cohortname_phenotype_analysis_date.txt**

Where:

Cohort: will be an identifier for the specific cohort eg NTR (Netherlands Twin Register)

Phenotype: is either DZT or MOTHDZT or MZT

Analysis: chromosome number (i.e. “chr1”) if files are provided by chromosome or (“all”) if all chromosomes included in one file

Date: will be the date of the day of the uploading (ddmmyy)

For example a file name from our cohort would be:

***NTR_MOTHDZT_all_151017***

*For monomorphic SNPs, report all SNPs that were monomorphic in your population in a separate txt‐format file with the following columns:

- **chr**: chromosome number
- **position:** basepair position according to Build37
- **markersnp**: Please use the markername exactly as it is represented in the imputation output (Build37). We will convert these to a common Id during the cleaning and meta-analysis.

File name should be:

**monomorphic_cohortname_date.txt**

where:

cohortname will be an identifier for the specific cohort

date**:** will be the date of the day of the uploading (ddmmyy)

**Please, always compress files before uploading! (use gzip)**

1. Data exchange procedure

GWAS results will be uploaded to the ShareSpaces file sharing system.

Details for the username and password will be provided later.

Any question please email :

Hamdi Mbarek (hmbarek@vu.nl) or Dorret Boomsma ([di.boomsma@vu.nl](mailto:di.boomsma@vu.nl))

**Summary**

- **Files required:**

1/ GWAS DZvsCTRL or MOTHDZTvsCTRL

2/ GWAS MZvsCTRL

For MOTHDZTvsCTRL please provide separate analyses one with full data and one excluding this group if you have larger N of Mothers who received fertility treatment.

- **Deadline dates**:

1/ Study Description by **01 December 2017**

2/ GWAS results by **15 December 2017**
