# Supplementary material for: Genome-wide association study meta-analysis of dizygotic twinning illuminates genetic regulation of female fecundity
Source: Hum Reprod. 2023 Dec 5;39(1):240–57. doi: 10.1093/humrep/dead247 (PMC10767824; doi:10.1093/humrep/dead247)
Supplement: dead247_Supplementary_Figure_S1 [file dead247_supplementary_figure_s1.pdf]

OXFORD
human reproduction

Human Reproduction, 2023, 1–1
https://doi.org/10.1093/humrep/dead247
Supplementary Data

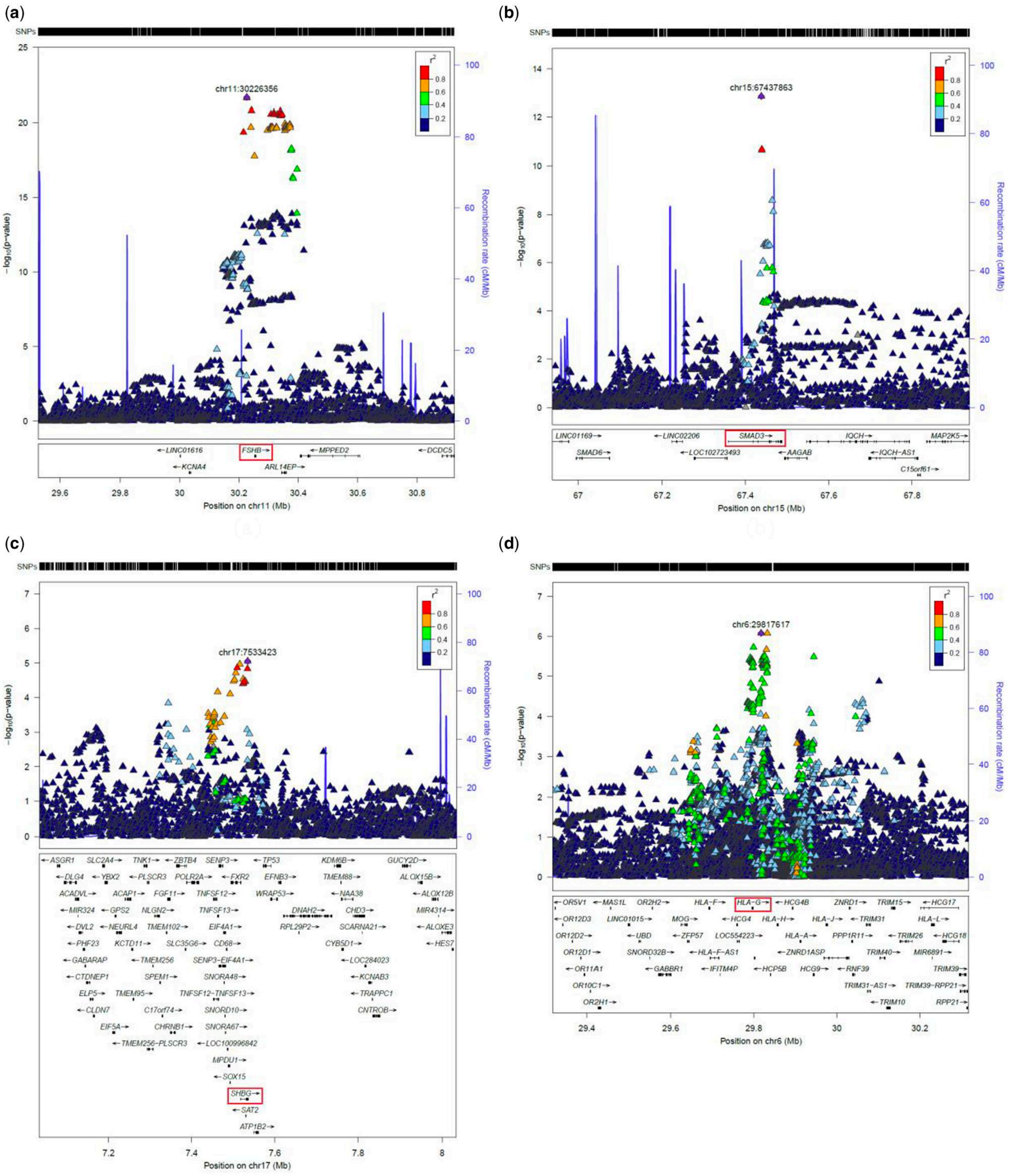

Supplementary Figure S1. Regional association plots for previously reported and below-significance regions. Regional association plots for four regions in Table 2, either previously reported (11p14.1 and 15q22.23 peaks) or reaching suggestive levels only (17p13.1 and 6p22.1 peaks). The reference SNP is the most highly associated one reported. The suggested gene in the text is highlighted in red. Loci are: (**a**) 11p14.1; (**b**) 15q22.33; (**c**) 17p13.1; (**d**) 6p22.1. Plots were prepared using a stand-alone copy of LocusZoom 1.3 (http://locuszoom.org/) for the 1000G Release 3 LD reference and hg19/Build 37.
