# Supplementary material for: Genome-wide association study meta-analysis of dizygotic twinning illuminates genetic regulation of female fecundity
Source: Hum Reprod. 2023 Dec 5;39(1):240–57. doi: 10.1093/humrep/dead247 (PMC10767824; doi:10.1093/humrep/dead247)
Supplement: dead247_Supplementary_Figure_S2 [file dead247_supplementary_figure_s2.pdf]

OXFORD
human reproduction

Human Reproduction, 2023, 1–1
https://doi.org/10.1093/humrep/dead247
Supplementary Data

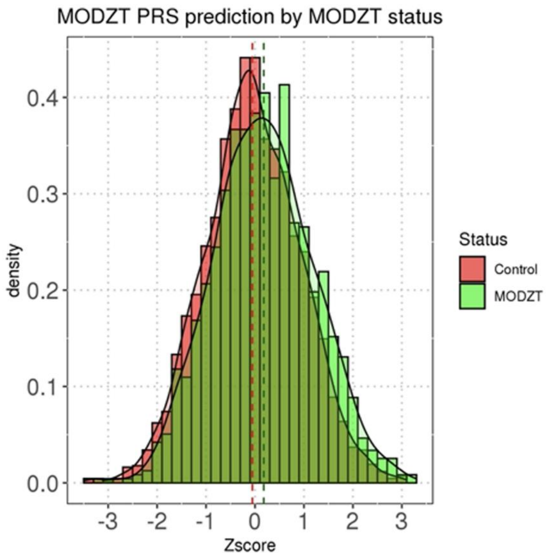

Supplementary Figure S2. DZ twinning PRS distributions in the NTR. Histograms for the distributions of PRS prediction in NTR MoDZT cases and controls. PRS were calculated from a GWAMA of discovery samples excluding the NTR data. The PRS with genome-wide significance of $P = 0.001$ was most highly correlated with case status and was used to independently predict DZ twinning in the target NTR sample (1186 MoDZT cases, 337 controls).
