# Supplementary material for: Genome-wide association study meta-analysis of dizygotic twinning illuminates genetic regulation of female fecundity
Source: Hum Reprod. 2023 Dec 5;39(1):240–57. doi: 10.1093/humrep/dead247 (PMC10767824; doi:10.1093/humrep/dead247)
Supplement: dead247_Supplementary_Figure_S3 [file dead247_supplementary_figure_s3.pdf]

OXFORD
human reproduction
Human Reproduction, 2023, 1–1
https://doi.org/10.1093/humrep/dead247
Supplementary Data

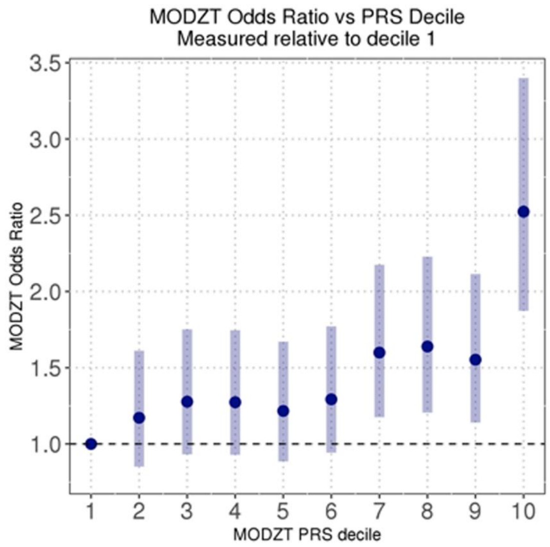

Supplementary Figure S3. Odds ratios for DZ twinning in the NTR by PRS decile. The PRS was constructed from the GWAMA of MoDZT and DZT, excluding the NTR data. The *P*-value threshold of 0.001 was used for prediction in the NTR dataset. Odds ratios and 95% confidence intervals were estimated with logistic regression including 10 PCs. The reported odds ratios are relative to the first decile. The points represent odds ratios and the bars represent the lower and upper 95% CI of the odds ratios.
