# Supplementary material for: Genome-wide association study meta-analysis of dizygotic twinning illuminates genetic regulation of female fecundity
Source: Hum Reprod. 2023 Dec 5;39(1):240–57. doi: 10.1093/humrep/dead247 (PMC10767824; doi:10.1093/humrep/dead247)
Supplement: dead247_Supplementary_Figure_S4 [file dead247_supplementary_figure_s4.pdf]

*Human Reproduction*, 2023, 1–1
https://doi.org/10.1093/humrep/dead247
Supplementary Data

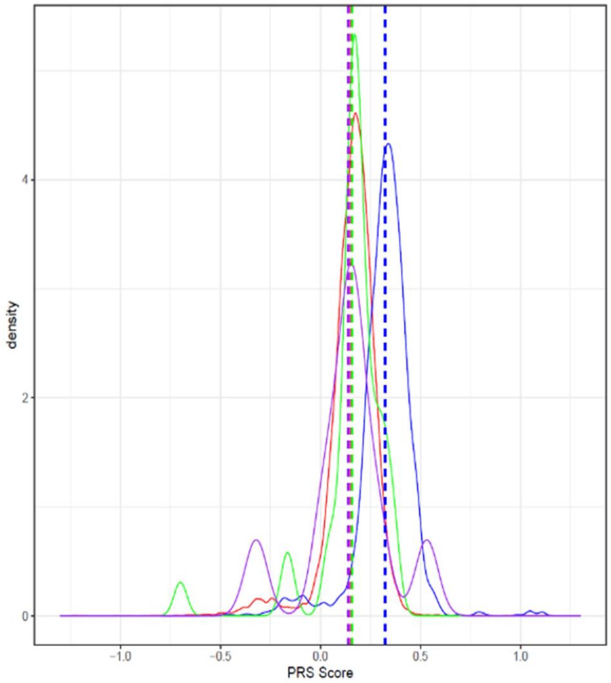


**Supplementary Figure S4. Distribution of DZ twinning PRS in out-of-sample Australian cases and controls and DZT pedigrees from Belgium and Utah.** PRS of Australian cases (blue, N = 1882, mean = 0.14, sd = 0.32) and controls (red, N = 15 252, mean = 0.16, sd = 0.14) compared with 40 cases from 10 multiplex families from Belgium (green) and 10 cases from 2 multiplex families from Utah (purple). The mean PRS of cases from multiplex families from Belgium and Utah (N = 50, mean = 0.16, sd = 0.18) is more similar to Australian controls.
