# Supplementary material for: Genome-wide association study meta-analysis of dizygotic twinning illuminates genetic regulation of female fecundity
Source: Hum Reprod. 2023 Dec 5;39(1):240–57. doi: 10.1093/humrep/dead247 (PMC10767824; doi:10.1093/humrep/dead247)
Supplement: dead247_Supplementary_Figure_S5 [file dead247_supplementary_figure_s5.pdf]

human
reproduction

Human Reproduction, 2023, 1–1
https://doi.org/10.1093/humrep/dead247
Supplementary Data

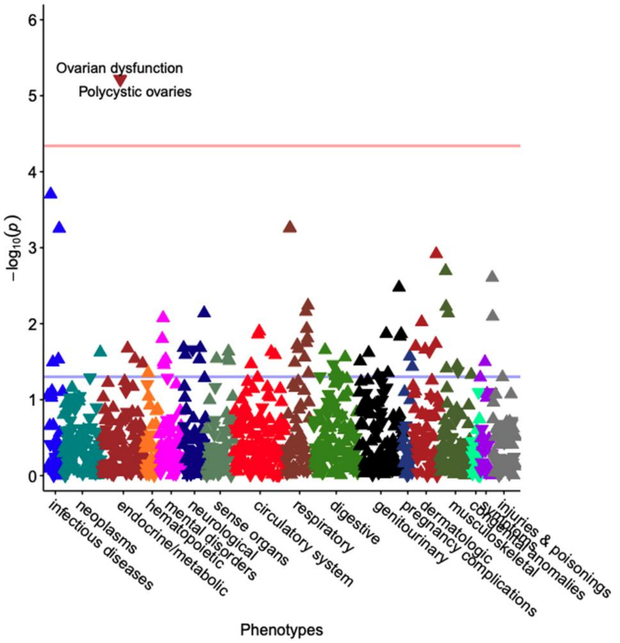

Supplementary Figure S5. PheWAS results for the DZ twinning PRS. Manhattan plot of the PheWAS for GWAMA-derived PRS and phenotypes in the BioVU Data. The -log10 of the *P*-value of the association between the PRS and the phenotype from a logistic regression model adjusted for age and the first 10 PCs is shown on the y-axis. Along the x-axis, phenotypes are colour-coded according to category. Triangles oriented upward represent beta values that are directionally concordant with the MoDZT and DZT PRS. Downward-oriented triangles have beta values in opposing directions between the phenotype and PRS. The red line is the Bonferroni corrected significance threshold ($\propto = 4.4 \times 10\text{–}^{05}$) and the blue line is the false discovery rate ($\propto = 0.05$).
