# Supplementary material for: Genome-wide association study meta-analysis of dizygotic twinning illuminates genetic regulation of female fecundity
Source: Hum Reprod. 2023 Dec 5;39(1):240–57. doi: 10.1093/humrep/dead247 (PMC10767824; doi:10.1093/humrep/dead247)
Supplement: dead247_Supplementary_Figure_S6 [file dead247_supplementary_figure_s6.pdf]

OXFORD
human reproduction
*Human Reproduction*, 2023, 1–1
https://doi.org/10.1093/humrep/dead247
Supplementary Data

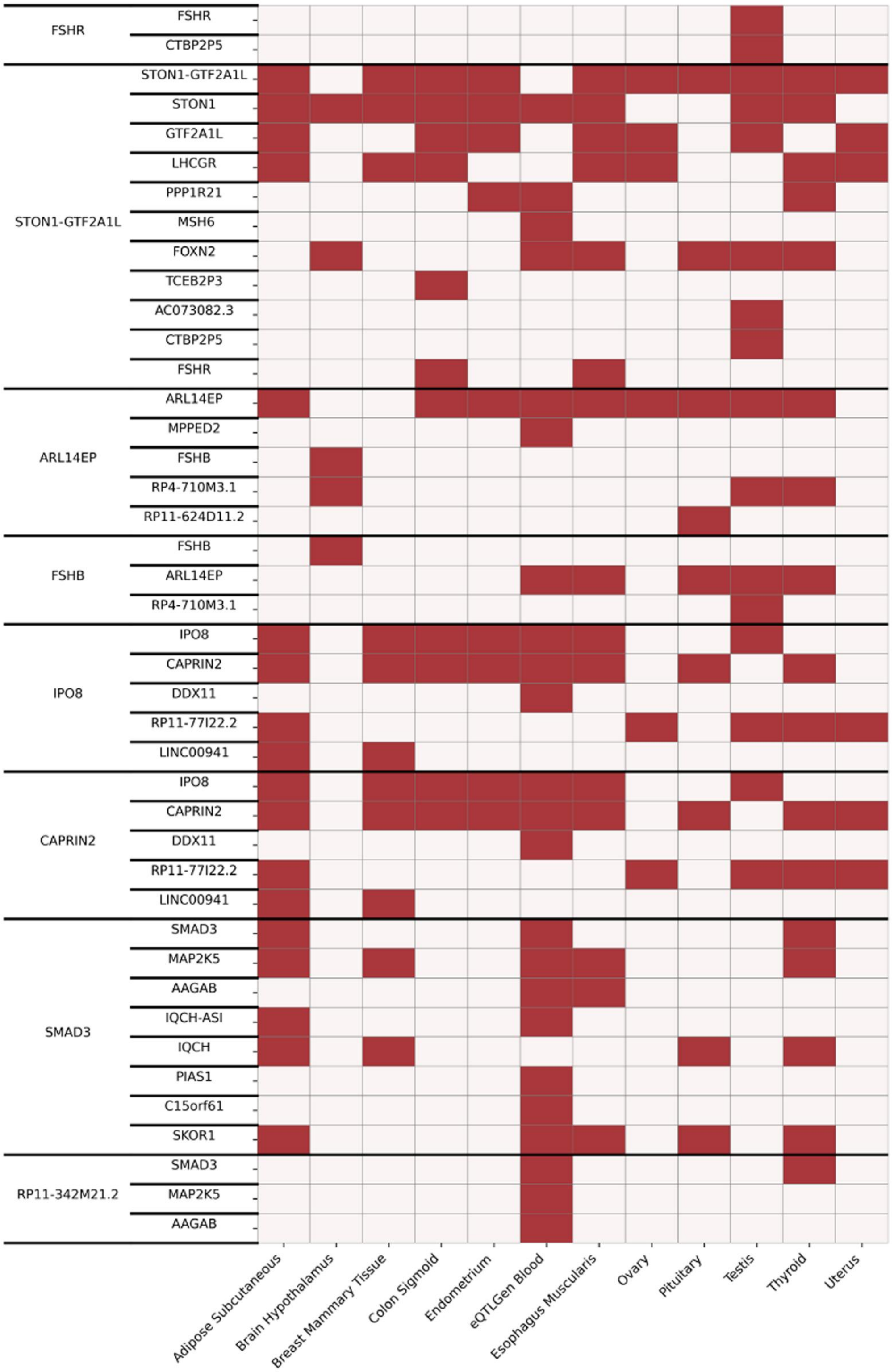


**Supplementary Figure S6. eQTLs in endometrium, blood and GTEx tissues that overlap SNPs in the gene-based association analysis.** SNPs annotated to genes in the gene-based association analysis have also been associated with the expression of several genes in multiple tissues as eQTLs. eQTL genes in each locus are shaded if their expression is significantly associated with a SNP from the gene-based association analysis in a given tissue. Alternating shades of grey define a new locus.
