# Supplementary material for: Genome-wide association study meta-analysis of dizygotic twinning illuminates genetic regulation of female fecundity
Source: Hum Reprod. 2023 Dec 5;39(1):240–57. doi: 10.1093/humrep/dead247 (PMC10767824; doi:10.1093/humrep/dead247)
Supplement: dead247_Supplementary_Figure_S7 [file dead247_supplementary_figure_s7.pdf]

OXFORD

human reproduction

Human Reproduction, 2023, 1–1

https://doi.org/10.1093/humrep/dead247

Supplementary Data

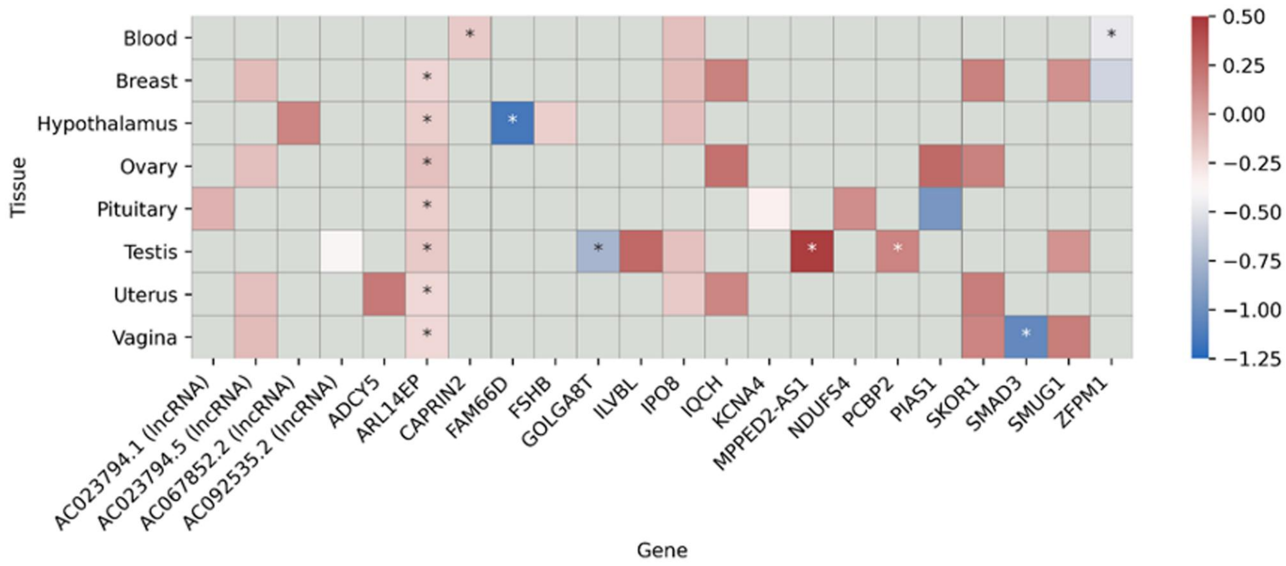

Supplementary Figure S7. Effect sizes of the significant genes for the TWAS for DZ twinning. Heatmap showing the effect sizes for significant genes and long non-coding RNAs (lncRNA) for the TWAS for DZ twinning after a Bonferroni correction per tissue. Darker shading corresponds to effect sizes of greater magnitude. Light gray squares indicate missing or unavailable data. Squares with a * indicate that the effect is significant after a Bonferroni correction accounting for the total number of tests across tissues (Supplementary Table S16). Gene names are displayed on the *x*-axis and tissue types on the y-axis. AC023794.1 lncRNA is part of the YRNA family. AC023794.5 lncRNA is in *SMUG1*. AC067852.2 lncRNA is known as HSD17B1 Antisense RNA 1. AC092535.2 lncRNA is near *CTBP1*.
