# Supplementary material for: Genome-wide association study meta-analysis of dizygotic twinning illuminates genetic regulation of female fecundity
Source: Hum Reprod. 2023 Dec 5;39(1):240–57. doi: 10.1093/humrep/dead247 (PMC10767824; doi:10.1093/humrep/dead247)
Supplement: dead247_Supplementary_Figure_S8 [file dead247_supplementary_figure_s8.pdf]

OXFORD
human reproduction
Human Reproduction, 2023, 1–1
https://doi.org/10.1093/humrep/dead247
Supplementary Data

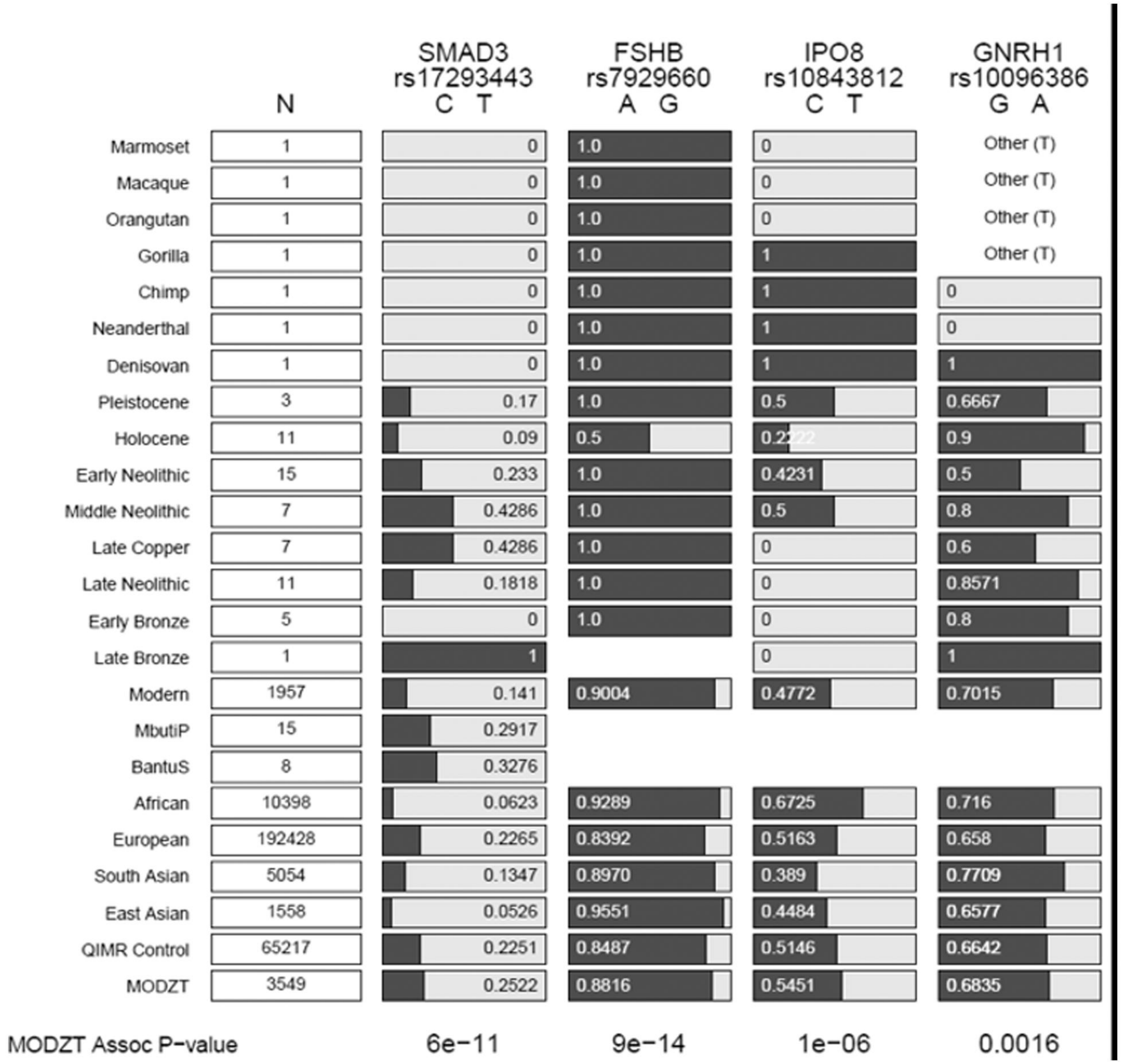

Supplementary Figure S8. Allele frequencies of four SNPs associated with DZ twinning in primates and ancient humans. The four SNPs were directly genotyped by Haak *et al.* (2005) from selected primates and DNA from archaic human samples. SNPs were selected based on being the best tag SNP of the associated genes in the present study. For each SNP, the first allele listed is that associated with DZ twinning. Note that the DZT associated SNPs in *ZNFP1* and *FSHR* were not well tagged on the Axiom array used by Haak.
