# Supplementary material for: Genome-wide association study meta-analysis of dizygotic twinning illuminates genetic regulation of female fecundity
Source: Hum Reprod. 2023 Dec 5;39(1):240–57. doi: 10.1093/humrep/dead247 (PMC10767824; doi:10.1093/humrep/dead247)
Supplement: dead247_Supplementary_Figure_S9 [file dead247_supplementary_figure_s9.pdf]

OXFORD

human reproduction

Human Reproduction, 2023, 1–1
https://doi.org/10.1093/humrep/dead247
Supplementary Data

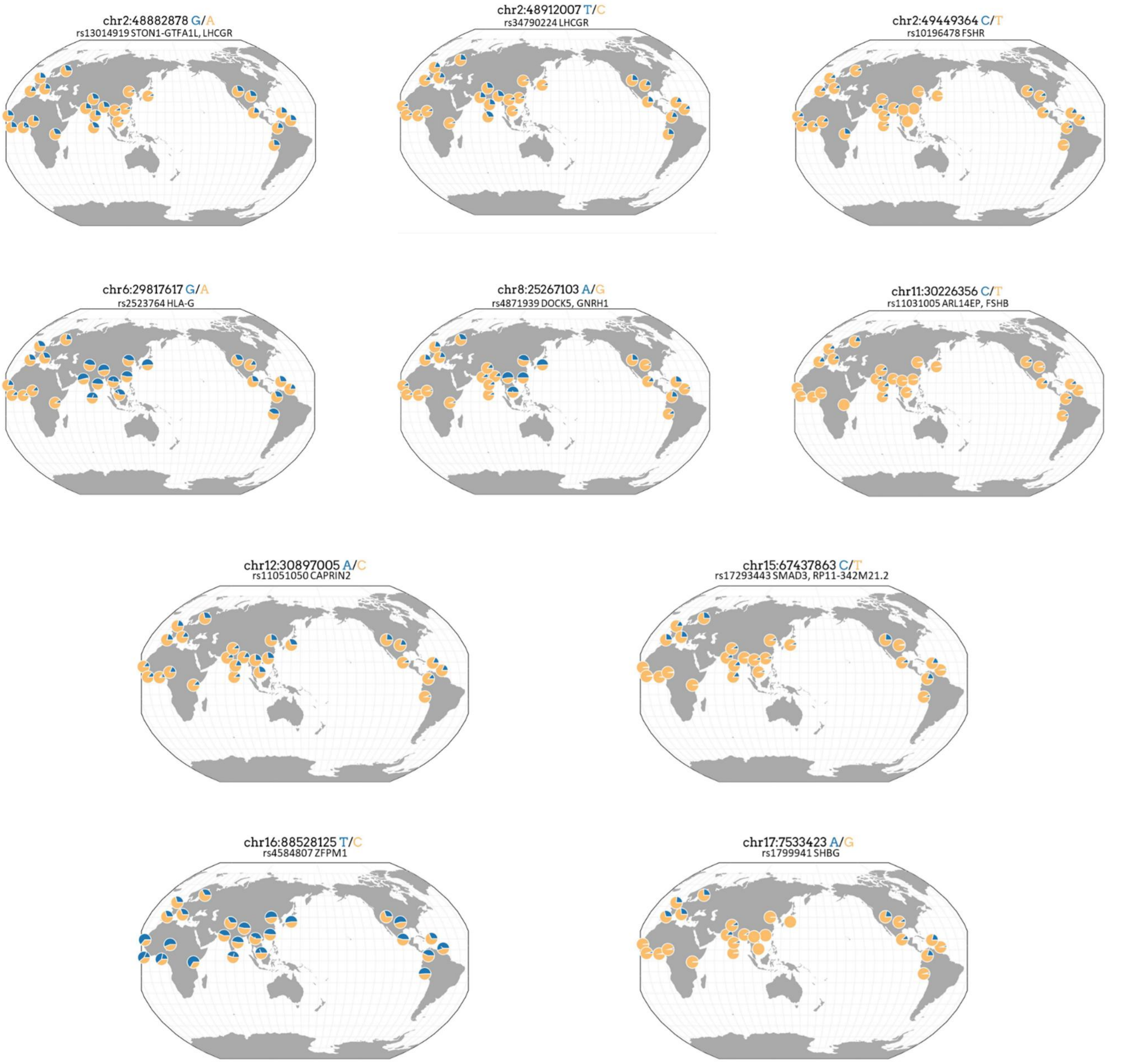

Supplementary Figure S9. Frequencies of top DZ twinning SNPs in global populations. For each DZ twinning associated gene and SNP, coloured wheels represent global population allele frequencies of the corresponding alleles listed above each image.
