# Supplementary material for: Genome-wide association study meta-analysis of dizygotic twinning illuminates genetic regulation of female fecundity
Source: Hum Reprod. 2023 Dec 5;39(1):240–57. doi: 10.1093/humrep/dead247 (PMC10767824; doi:10.1093/humrep/dead247)
Supplement: dead247_Supplementary_Figure_S10 [file dead247_supplementary_figure_s10.pdf]

Human Reproduction, 2023, 1–1
https://doi.org/10.1093/humrep/dead247
Supplementary Data

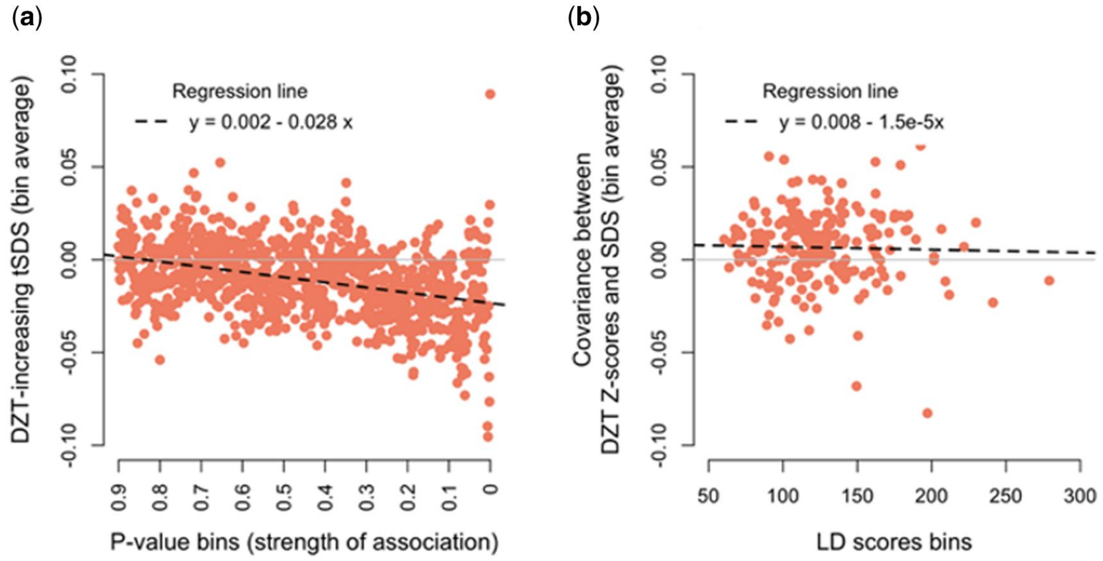

Supplementary Figure S10. Relationship between Singleton Density Scores (SDS) and DZ twinning association statistics. (**a**) The correlation between the strength of association between SNPs and DZT across 1000 *P*-value bins (*x*-axis: mean *P*-value in the bin; *y*-axis: mean SDS of DZT-increasing allele). The horizontal grey line ($y = 0$) represents the expectation under the null hypothesis of no polygenic selection of DZT alleles. (**b**) The relationship between LD scores (*x*-axis) and covariance between DZT association Z-scores and SDS (*y*-axis). Each dot represents a SNP bin defined by 1703 European ancestry LD blocks.
