# Supplementary material for: Genome-wide association study meta-analysis of dizygotic twinning illuminates genetic regulation of female fecundity
Source: Hum Reprod. 2023 Dec 5;39(1):240–57. doi: 10.1093/humrep/dead247 (PMC10767824; doi:10.1093/humrep/dead247)
Supplement: dead247_Supplementary_Table_S1 [file dead247_supplementary_table_s1.pdf]

OXFORD
human reproduction

Human Reproduction, 2023, 1–1
https://doi.org/10.1093/humrep/dead247
Supplementary Data

Supplementary Table S1. Overview on fertility information, genotyping array and imputation panel for each cohort.

Twin dataset	Information available on mode of twins conception: spontaneous (or before 1980) vs with ART	Genotyping array	Imputation reference panel: 1kG phase 1 v3 (March 2012)	1kG phase 3 v5	HRC v1	TopMed
NTR	MODZT screened for ART	Perlegen-Affymetrix, Affymetrix 6.0, Affymetrix Axiom, Illumina Human Quad Bead 660, Illumina Omni 1M and Illumina GSA			x	
QIMR	MODZT screened for ART	HapMap-derived (317K, 370, 610K, 660K); 1000 Genomes-derived (OmniExpress, Omni2.5, Core+Exome, PsychArray) and Global Screening Array (GSA v1)			x	
MCTFR	Twins were born in the 1970s or early 1980s	Illumina's Human 660W-Quad Array	x			
US WGHS	MODZT were of reproductive age before the common use of ART (born at latest in 1951)	HumanHap300 Duo '+' chips or the combination of the HumanHap300 Duo and iSelect chips (Illumina, San Diego, CA, USA) with the Infinium II protocol	x			
deCODE	Twins born before 1991	Illumina chips				
UK Biobank	Twins born before 1967	UK Biobank Axiom^{TM} Array				
Finland	Twins born before 1979	Illumina Human610-Quad v1.0 B, Human670-QuadCustom v1.0 A, Illumina HumanCoreExome- (12 v1.0 A, 12 v1.1 A, 24 v1.0 A, 24 v1.1 A, 24 v1.2 A) and Affymetrix FinnGen Axiom arrays				x
Sweden	Twins born before 1958	Illumina OmniExpress	x			
UK Twins	Twins born before 1967	Illumina HumanHap300 BeadChip and Illumina HumanHap610 QuadChip	x			
UK TEDS	Twins born before 1967	Affymetrix GeneChip 6.0 or Illumina HumanOmniExpressExome chips			x	
