# Supplementary material for: Genome-wide association study meta-analysis of dizygotic twinning illuminates genetic regulation of female fecundity
Source: Hum Reprod. 2023 Dec 5;39(1):240–57. doi: 10.1093/humrep/dead247 (PMC10767824; doi:10.1093/humrep/dead247)
Supplement: dead247_Supplementary_Table_S2 [file dead247_supplementary_table_s2.pdf]

Human Reproduction, 2023, 1–1
https://doi.org/10.1093/humrep/dead247
Supplementary Data

Supplementary Table S2. Genome-wide significant loci in the meta-analysis of mothers of spontaneous DZ twins.

SNP	Locus	Position	Gene	Annotation	Risk Allele	Risk Allele Frequency	Beta (SE)	P
rs4871939	8p21.2	25267103	DOCK5	intron	A	0.248	−0.08 (0.013)	9.06×10^{-10}
rs11031005	11p14.1	30226356	FSHB	5′ upstream	T	0.866	0.156 (0.016)	2.17×10^{-22}
rs17293443	15q22.33	67437863	SMAD3	intron	T	0.772	−0.098 (0.013)	1.39×10^{-13}
rs4584807	16q24.2	88528125	ZFPM1	intron	T	0.313	−0.069 (0.012)	4.39×10^{-8}
