# Supplementary material for: Genome-wide association study meta-analysis of dizygotic twinning illuminates genetic regulation of female fecundity
Source: Hum Reprod. 2023 Dec 5;39(1):240–57. doi: 10.1093/humrep/dead247 (PMC10767824; doi:10.1093/humrep/dead247)
Supplement: dead247_Supplementary_Table_S6 [file dead247_supplementary_table_s6.pdf]

OXFORD

human reproduction

Human Reproduction, 2023, 1–1
https://doi.org/10.1093/humrep/dead247
Supplementary Data

Supplementary Table S6. Chi-squared test results of Belgian cases (N = 21) versus controls (N = 47 567) for 12 SNPs in DZT genes identified from the gene-based test.

Gene	SNP	N	Allele	Case	Control	Chi-square	AsyP	EmpP	Iters	FDR
HLA-G	rs1611160	47 588	T	0.714	0.471	10.2	0.001	0.002	8649	0.0422
GNRH1	rs6185	38 877	G	0.071	0.226	4.8	0.028	0.031	652	0.2329
GNRH1	rs4871939	39 610	A	0.071	0.223	4.7	0.03	0.042	478	0.1744
HLA-G	rs2523764	47 546	G	0.5	0.339	4.6	0.032	0.04	499	0.1302
CAPRIN2	rs11051050	46 684	A	0.357	0.239	2.9	0.088	0.145	138	0.2839
IPO8	rs10771757	46 050	T	0.35	0.235	2.7	0.101	0.128	156	0.2924
FSHR	rs12473870	47 338	A	0.524	0.407	2.3	0.126	0.149	134	0.2426
LHCGR	rs34790224	46 474	T	0.3	0.21	1.8	0.183	0.345	58	0.2532
SHBG	rs1799941	47 533	A	0.333	0.257	1.2	0.274	0.238	84	0.3457
SMAD3	rs17293443	65 534	C	0.286	0.227	0.8	0.376	0.345	58	0.6667
ARL14EP	rs4071559	46 951	T	0.119	0.151	0.4	0.549	0.435	46	0.5682
ZFPM1	rs4584807	42 959	T	0.269	0.311	0.2	0.644	0.588	34	1
FSHB	rs506306	47 543	G	0.429	0.426	0	0.974	1	20	1
STON1-GTF2A1L	rs13014919	46 666	G	0.29	0.275	0	0.845	0.8	25	1
ARL14EP	rs11031005	46 500	C	0.143	0.133	0	0.845	1	20	1
FSHR	rs10196478	45 200	C	0.111	0.116	0	0.933	0.87	23	1

Overall test of association with these SNPs gives Fisher $P = 0.0137$.
