# Supplementary material for: Genome-wide association study meta-analysis of dizygotic twinning illuminates genetic regulation of female fecundity
Source: Hum Reprod. 2023 Dec 5;39(1):240–57. doi: 10.1093/humrep/dead247 (PMC10767824; doi:10.1093/humrep/dead247)
Supplement: dead247_Supplementary_Table_S9 [file dead247_supplementary_table_s9.pdf]

OXFORD
human reproduction
Human Reproduction, 2023, 1–1
https://doi.org/10.1093/humrep/dead247
Supplementary Data

Supplementary Table S9. Summary of the genetic correlations (rg) between dizygotic twinning and *in vivo* hormones levels.

Trait	Trait 2	rg	*P*-value
DZ twinning	Testosterone (quantile) female	−0.200 (0.0907)	0.0276
DZ twinning	Testosterone (quantile) male	0.133 (0.0820)	0.104
DZ twinning	SHBG (quantile) both sexes	0.0950 (0.0715)	0.184
DZ twinning	SHBG (quantile) male	0.0922 (0.0706)	0.191
DZ twinning	SHBG (quantile) female	0.0997 (0.0962)	0.3
DZ twinning	Free Testosterone	0.111 (0.108)	0.304
DZ twinning	Oestradiol (quantile) male	−0.159 (0.255)	0.534
DZ twinning	Oestradiol (quantile) both sexes	−0.0637 (0.189)	0.737
DZ twinning	Oestradiol (quantile) female	−0.0115 (0.265)	0.965
DZ twinning	Testosterone (quantile) both sexes	−0.000761 (0.0823)	0.993
