# Supplementary material for: Genome-wide association study meta-analysis of dizygotic twinning illuminates genetic regulation of female fecundity
Source: Hum Reprod. 2023 Dec 5;39(1):240–57. doi: 10.1093/humrep/dead247 (PMC10767824; doi:10.1093/humrep/dead247)
Supplement: dead247_Supplementary_Table_S10 [file dead247_supplementary_table_s10.pdf]

OXFORD
human reproduction
Human Reproduction, 2023, 1–1
https://doi.org/10.1093/humrep/dead247
Supplementary Data

Supplementary Table S10. Summary of the genetic correlations (rg) between dizygotic twinning, cancers and smoking.

Trait	Trait 2	rg	P-value
DZ twinning	Lung cancer	0.3	0.009
DZ twinning	Illness of father: Lung cancer	0.34	8.94 e−3
DZ twinning	Illness of mother: Lung cancer	0.62	0.03
DZ twinning	Bowel cancer	0.559	0.02
DZ twinning	Prostate cancer	0.18	0.03
DZ twinning	Cigarettes smoked per day	0.45	0.0018
DZ twinning	Ever smoked	0.25	0.015
