# Supplementary material for: Genome-wide association study meta-analysis of dizygotic twinning illuminates genetic regulation of female fecundity
Source: Hum Reprod. 2023 Dec 5;39(1):240–57. doi: 10.1093/humrep/dead247 (PMC10767824; doi:10.1093/humrep/dead247)
Supplement: dead247_Supplementary_Table_S15 [file dead247_supplementary_table_s15.pdf]

OXFORD

human reproduction

Human Reproduction, 2023, 1–1

https://doi.org/10.1093/humrep/dead247

Supplementary Data

Supplementary Table S15. TWAS: the number of genes included in each multi-tissue model and the corresponding Bonferroni correction.

Tissue	N genes	Bonferroni correction
Blood	10299	4.85E−06
Breast	13050	3.83E−06
Hypothalamus	8538	5.86E−06
Ovary	8676	5.76E−06
Pituitary	11119	4.50E−06
Testis	16645	3.00E−06
Uterus	7098	7.04E−06
Vagina	7254	6.89E−06
Combined	82679	6.74E−07
